# EzColocalization: An ImageJ plugin for visualizing and measuring colocalization in cells and organisms

**DOI:** 10.1038/s41598-018-33592-8

**Published:** 2018-10-25

**Authors:** Weston Stauffer, Huanjie Sheng, Han N. Lim

**Affiliations:** 1 Department of Integrative Biology, University of California Berkeley, Berkeley, CA USA; 2 Present Address: Atomwise Inc., San Francisco, CA USA

## Abstract

Insight into the function and regulation of biological molecules can often be obtained by determining which cell structures and other molecules they localize with (*i.e*. colocalization). Here we describe an open source plugin for ImageJ called EzColocalization to visualize and measure colocalization in microscopy images. EzColocalization is designed to be easy to use and customize for researchers with minimal experience in quantitative microscopy and computer programming. Features of EzColocalization include: (i) tools to select individual cells and organisms from images; (ii) filters to select specific types of cells and organisms based on physical parameters and signal intensity; (iii) heat maps and scatterplots to visualize the localization patterns of reporters; (iv) multiple metrics to measure colocalization for two or three reporters; (v) metric matrices to systematically measure colocalization at multiple combinations of signal intensity thresholds; and (vi) data tables that provide detailed information on each cell in a sample. These features make EzColocalization well-suited for experiments with low reporter signal, complex patterns of localization, and heterogeneous populations of cells and organisms.

## Introduction

Advances in microscopy equipment and labeling techniques make it possible for researchers to image a variety of biological molecules in almost any cell, tissue, or organism^1–7^. However, researchers often find it difficult to rigorously evaluate and interpret the images. In particular, it is often challenging to determine whether the different molecules of interest occur in the same locations, different locations or independent locations (*i.e*. colocalization, anticolocalization and noncolocalization respectively) in cells, tissues or organisms^8^.

Several factors limit the use of current software for visualizing the localization of reporters in biological samples and measuring colocalization^9–12^. One factor is that customization of the software is often required for the equipment, reporters and samples^13,14^, and for automated analyses. A second factor is that the software is often not suited to experiments that push the limits of detection, where the intensity of the intracellular signal is similar to the extracellular signal (*i.e*. “background”)^15^, and where there are high levels of non-specific signal in cells^8^. The latter can occur because the probes or reporters are not sufficiently specific^16^, are not adequately removed from cells or organisms^17^, or have low signal relative to endogenous compounds (*i.e*. “autofluorescence”)^18^. That is, software tools are needed to distinguish intracellular pixels from extracellular pixels, and to select signal intensity thresholds to limit analyses to a subset of intracellular pixels. A third factor is that there are often mixed localization patterns within cells and different localization patterns among cells in a sample^8,11,19^. When this heterogeneity is present, software is need to provide measurements for each cell or defined subsets of cells in samples.

It is often possible to address the above challenges by combining multiple existing software programs and customizing them^8,15^. However, combining and customizing software requires proficiency in programming, experience with quantitative microscopy, comfort with mathematics and statistics, and other support. Many researchers do not have these skills or resources, and this is a likely reason that many studies evaluate colocalization by the simple, but often misleading, method of overlaying red and green color images^10,11^. Therefore there is a pressing need for a single application that provides all the tools for start to finish analysis of colocalization and can be easily customized.

In this study, an open source plugin for ImageJ called EzColocalization was developed so that researchers at all levels of proficiency can visualize the localization of signals and measure colocalization via an easy-to-use graphical user interface (GUI). The first part of the study describes EzColocalization, and the second part demonstrates its use for different sample types and for resolving common issues that prevent rapid and robust quantitative measurements of colocalization. EzColocalization can measure colocalization in cells, tissues, and whole organisms (*e.g. Caenorhabditis elegans* and *Drosophila* embryos); and the software is especially helpful where automation and customization is required, to obtain individual cell measurements in samples with many cells, and for reporters with low signal or low specificity.

## Methods and Materials

### EzColocalization development

The code for EzColocalization was written in Eclipse Java Integrated Development Environment (IDE) release 4.3.0^20^, which is a workspace for writing code and detecting compiling errors in Java^TM^. EzColocalization incorporates ImageJ Application Program Interfaces (APIs) available from the National Institutes of Health, U.S. Department of Health and Human Services. An environment builder was used so that code written in the IDE ran in an instance of ImageJ as a plugin. This builder was implemented with Java Development Kit 8^21^ and the ImageJ source code within the IDE. The WindowBuilder^22^ plugin for the IDE was used to design and generate the code for the GUI, and the code produced was restructured and revised to improve readability, and add listeners, which obtain user inputs from the GUI for running the plugin.

The basic level of organization of the code for EzColocalization are “classes”. Classes are separated blocks of code that represent a set of methods and variables; a class may be devoted to performing calculations which share code or calculations that are most conveniently performed together. Classes with related operations are grouped into a higher level of organization termed “packages”. For example, a class that generates heat maps and a class that displays heat maps may be bundled into the same package. The classes and packages are described in detail in the Supplementary Information. Many processes within EzColocalization are performed as background computing, and thus the results of some classes, which are intermediates in longer methods, are not displayed and cannot be interacted with via the GUI.

### Testing of EzColocalization

**Figure 1:**
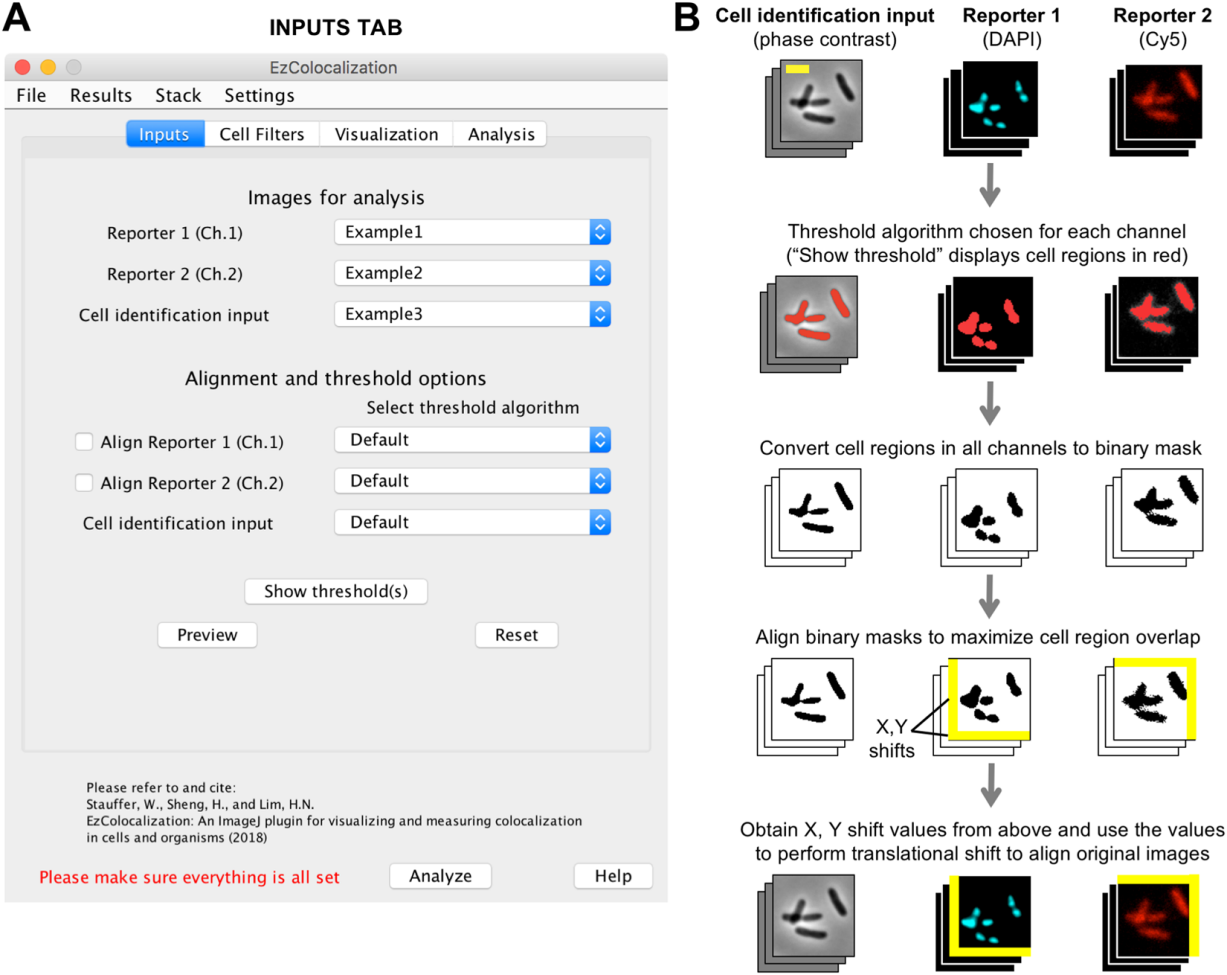
Inputs and alignment tab. (**A**). Inputs tab in the GUI. (**B**) General steps for the alignment of images. The cell identification image stack (phase contrast; left column), reporter 1 image stack (DAPI staining of DNA; center column), and reporter 2 image stack (Cy5; right column) are images of a previously reported bacterial strain (HL6320)^15^. Scale bar is 2 μm. Reporters 1 and 2 images are pseudocolored. Red coloring in the second row of images indicates the objects identified by thresholding of the signal in each channel (“Default” algorithm in ImageJ). Following alignment of the images, pixels that overhang are removed and gaps are filled with pixels with zero value (yellow areas) so that all images have the same area in the common aligned region.

**Figure 2:**
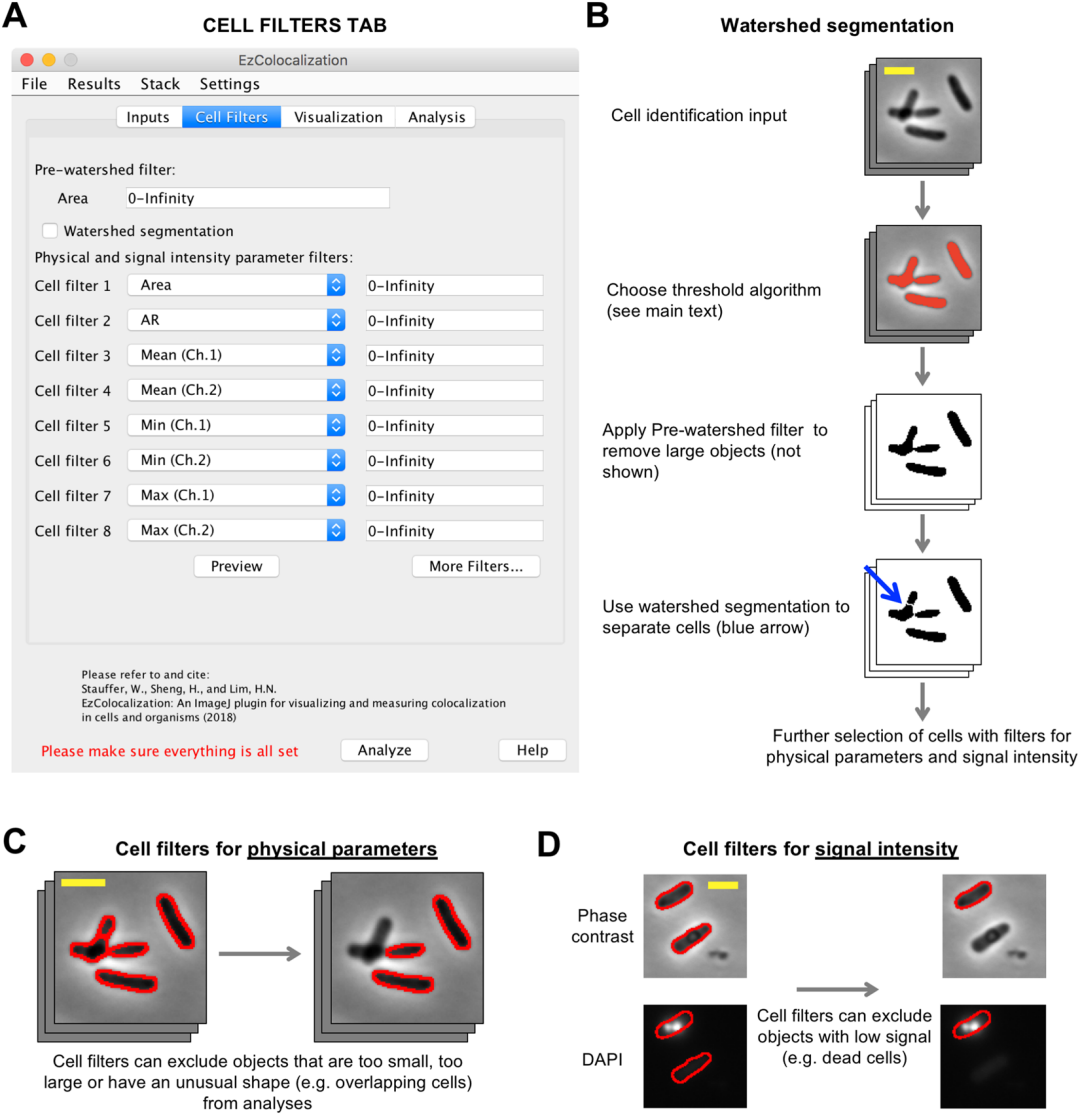
Cell identification and cell filters tab. (**A**) Cell Filters tab in the GUI. (**B**) Cell selection and watershed segmentation. Red coloring in the image in the second row indicates objects identified by thresholding of the signal in the cell identification channel (“Default” algorithm in ImageJ). Cells are the same as in Fig. 1. (**C**) Selection of cells based on physical features using the cell filters. Scale bar is 2 μm. Phase contrast image from Fig. 1. Red outline indicates the objects that were identified by thresholding (Panel B), and in the case of the right image, are within the parameter range(s) selected by the filter. (**D**) Selection of cells based on signal intensity using the cell filters. Phase contrast (cell identification image) and DAPI stain (reporter channel) images of bacteria (HL6187). Scale bar is 2 μm. Note: the lower of the two cells (no red border) has been removed from the analysis by the cell filter (that is, it no longer has the red cell outline).

**Figure 3:**
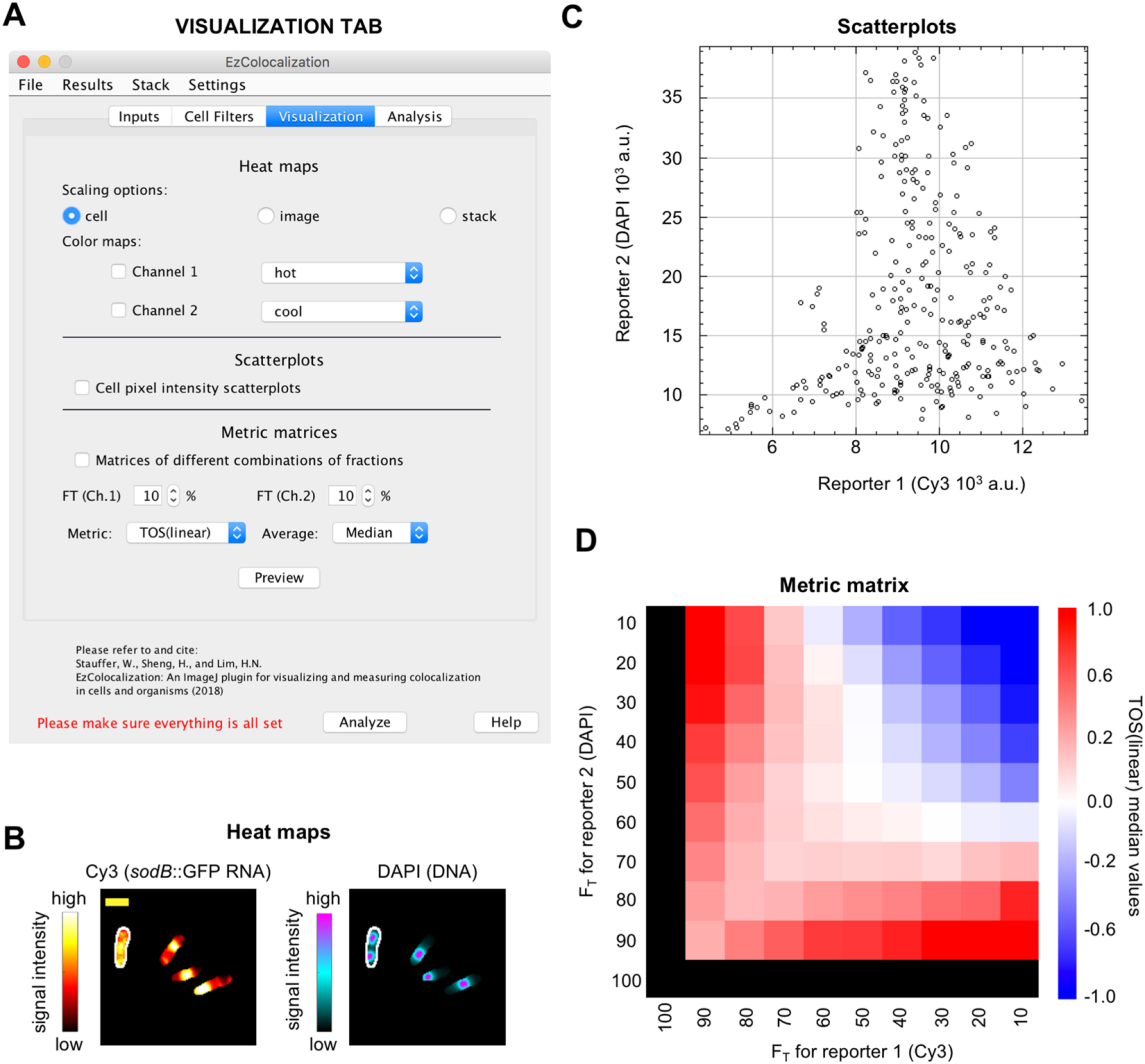
Visualization tab. Data are from bacteria (HL6187) with labeled *sodB::gfp* RNA (Cy3 channel) and DNA (DAPI). (**A**) Visualization tab in the GUI. (**B**) Heat maps of Cy3 and DAPI signals for bacteria with “cell scaling” (defined in main text). Scale bar is 2 μm. (**C**) Scatterplot of Cy3 and DAPI for the cell on the left and outlined in white in Fig. 3B. (**D**) Metric matrix for TOS (linear scaling) for the cell on the left and outlined in white in Fig. 3B. F_T_ is the top percentage of pixels in the channel; for example, if F_T_ for Cy3 is 80% then it refers to the 80% of pixels with the highest Cy3 signal. Black color on the left column and bottom row indicate that TOS values are not informative when one threshold is 100%; that is, the overlap of two reporters can only be 100% if 100% of pixels are selected for at least one channel.

**Figure 4:**
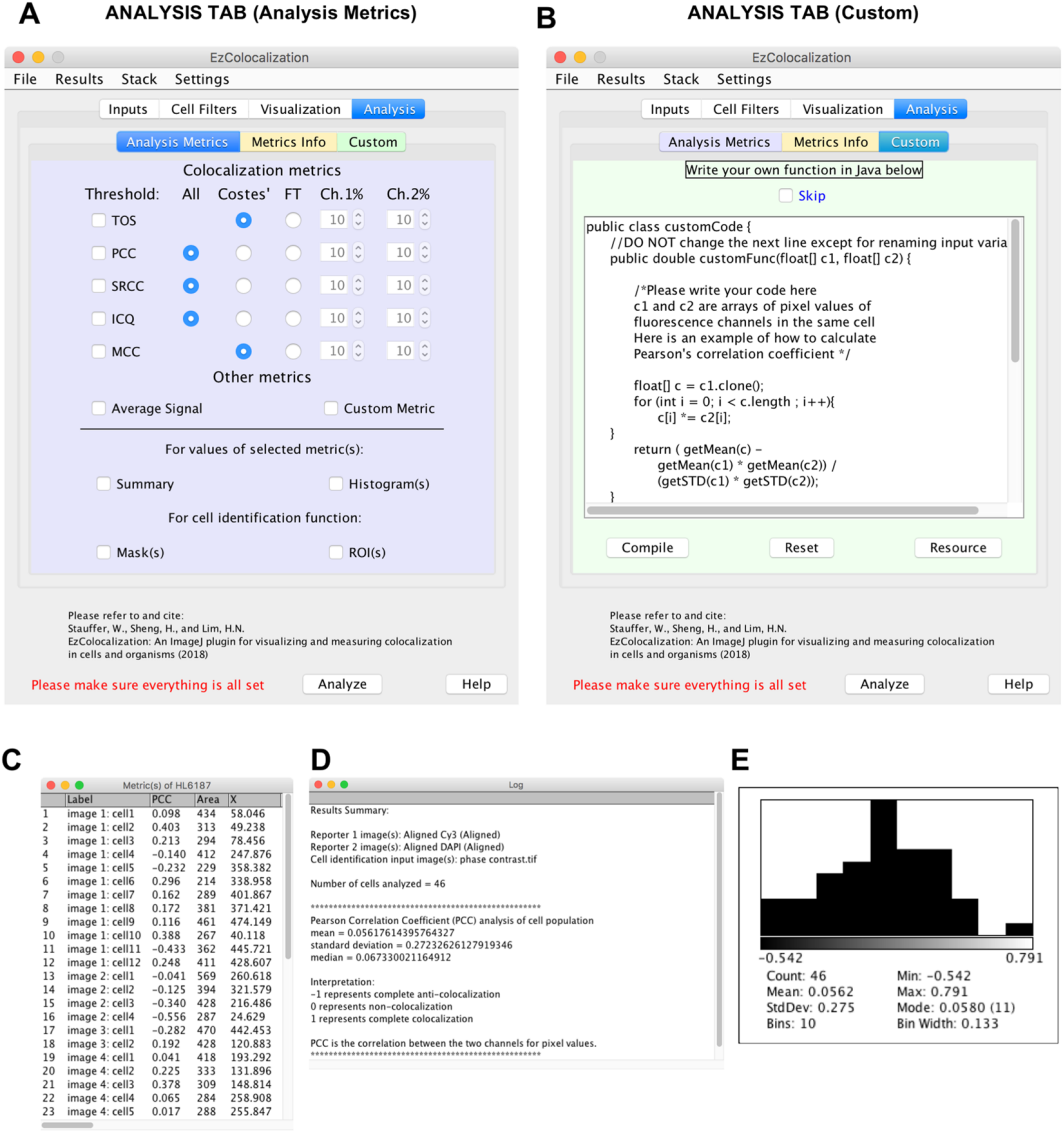
Analysis tab. (**A**) Analysis tab in the GUI for selecting default metrics. Note: this example is for two reporter channels (see Fig. 8G for 3 reporter channels). (**B**) Analysis tab in the GUI for users to code custom metrics. The example code provided is for measuring colocalization by Pearson correlation coefficient. (**C**) Example of a data table showing metric values for Pearson correlation coefficient (PCC) and some of the parameter values for some of the cells in the analysis. Label = the image and unique cell number to identify individual cells; Area = area of each cell in pixels; and X = the average x-value of all pixels in a cell. Data is from the example used in Fig. 3. (**D**) Summary report (“Log”) of the results in Fig. 4C. (**E**) Histogram generated from the results in Fig. 4C. The height of each bin is the relative frequency. The Count is the number of cells. Mean is the mean value. StdDev is the standard deviation. Bins is the number of bins. Min and Max are the minimum and maximum values of the lowest and highest bin respectively (which are shown immediately under the histogram). Mode is the mode value. Bin Width is the width of each bin within the histogram.

**Figure 5:**
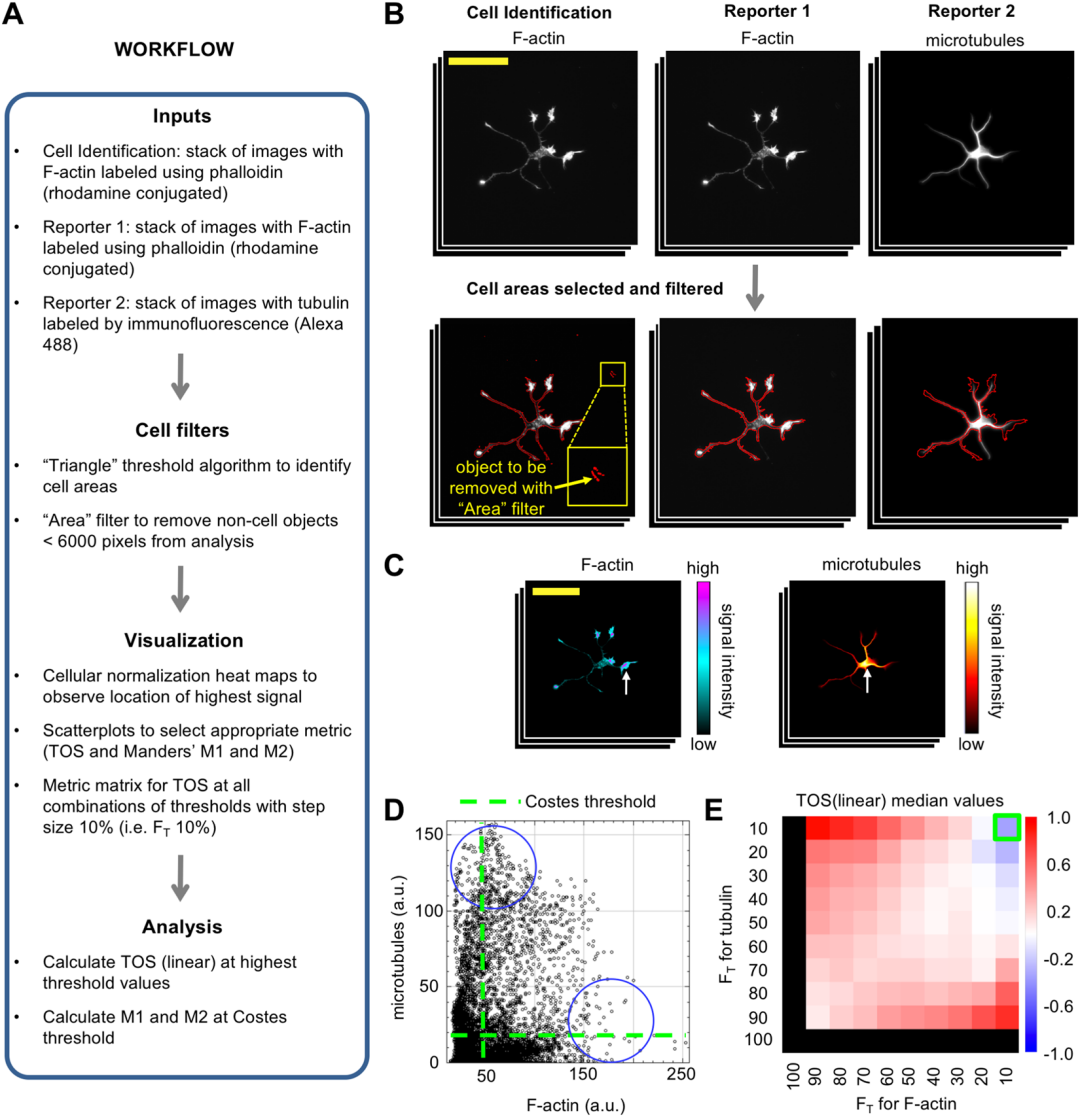
Application 1: Cell selection using reporter images and physical parameters. Images are rat hippocampal neurons labelled with an F-actin probe and anti-tubulin antibody visualized by fluorescence microscopy (see main text). (**A**) Workflow of the analysis. (**B**) Cell identification using the F-actin reporter and filters to remove small non-cell objects (yellow arrow) based on their size (*i.e*. Area option from the cell filters). Large yellow box in left panel is a zoomed in view of the smaller yellow box. Red outline of the neuron indicates it has been identified as an object (*i.e*. a cell) for analysis. Scale bar is 100 μm. (**C**) Heat maps with cellular normalization showing localization regions of signal intensity for the cell shown in panel B. Scale bar is the same as panel B. (**D**) Scatterplot showing relationship between the signal intensity for two reporter channels for a random cell in the sample. Pixels with the highest intensity signal for each reporter channel have the lowest intensity signals for the other reporter, which indicates anticolocalization (blue circles). Green dash lines indicate thresholds selected by Costes’ method. (**E**) Metric matrix for the median TOS (linear) value for all cells in the sample (*n* = 20). Green box indicates the threshold combination where F-actin and tubulin have the highest intensity signal (top 10% of pixels for each channel); the median TOS value is −0.36.

**Figure 6:**
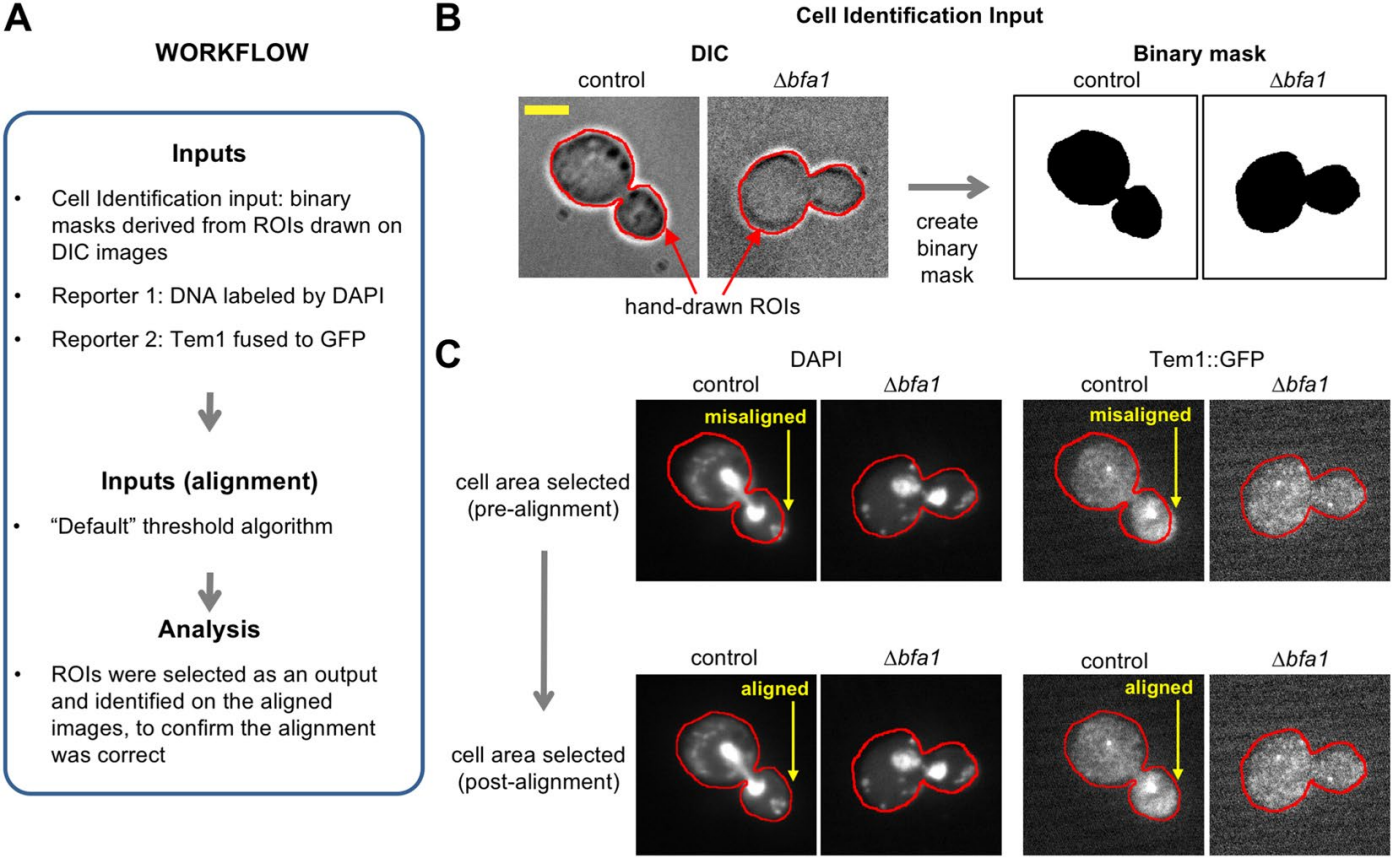
Application 2: Image alignment. Images are *S. cerevisiae* with TEM1 translationally fused to GFP and DAPI staining visualized by DIC microscopy and fluorescence microscopy (see main text). (**A**) Workflow of the analysis. (**B**) Cell identification by hand-drawn ROIs on a DIC image and creation of a binary image mask. Red outline indicates the boundary of the hand-drawn ROI. Scale bar is 3.5 μm. (**C**) Alignment of the reporter images using the binary mask image. Arrows indicate areas of misalignment that are corrected. Red outline is the same as for Panel B.

**Figure 7:**
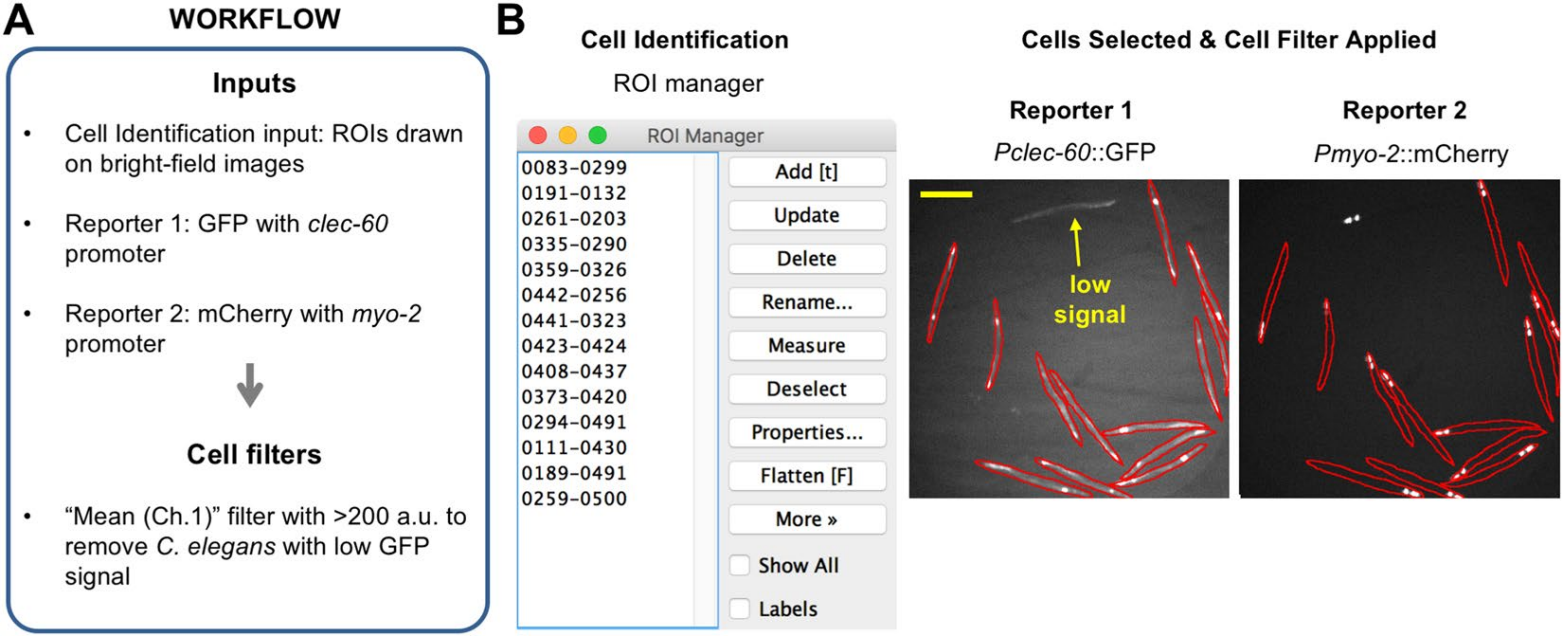
Application 3: Cell selection using signal intensity parameters. Images are whole adult *C. elegans* with GFP expressed from the *clec-60* promoter and mCherry expressed from the *myo-2* promoter that are visualized by bright-field microscopy and fluorescence microscopy (see main text). (**A**) Workflow of the analysis. (**B**) Selection of *C. elegans* so that only those individuals with an average intensity for the reporter signal that is above a threshold level are included in analyses. Left image shows the ROI manager with a list of ROIs that were hand-drawn around each *C. elegans*. Right image shows the reporter channel images with red outlines indicating the boundaries of the ROIs. *C. elegans* below the threshold level were excluded (yellow arrow) from the analyses by using the cell filters for signal intensity. Scale bar is 250 μm.

**Figure 8:**
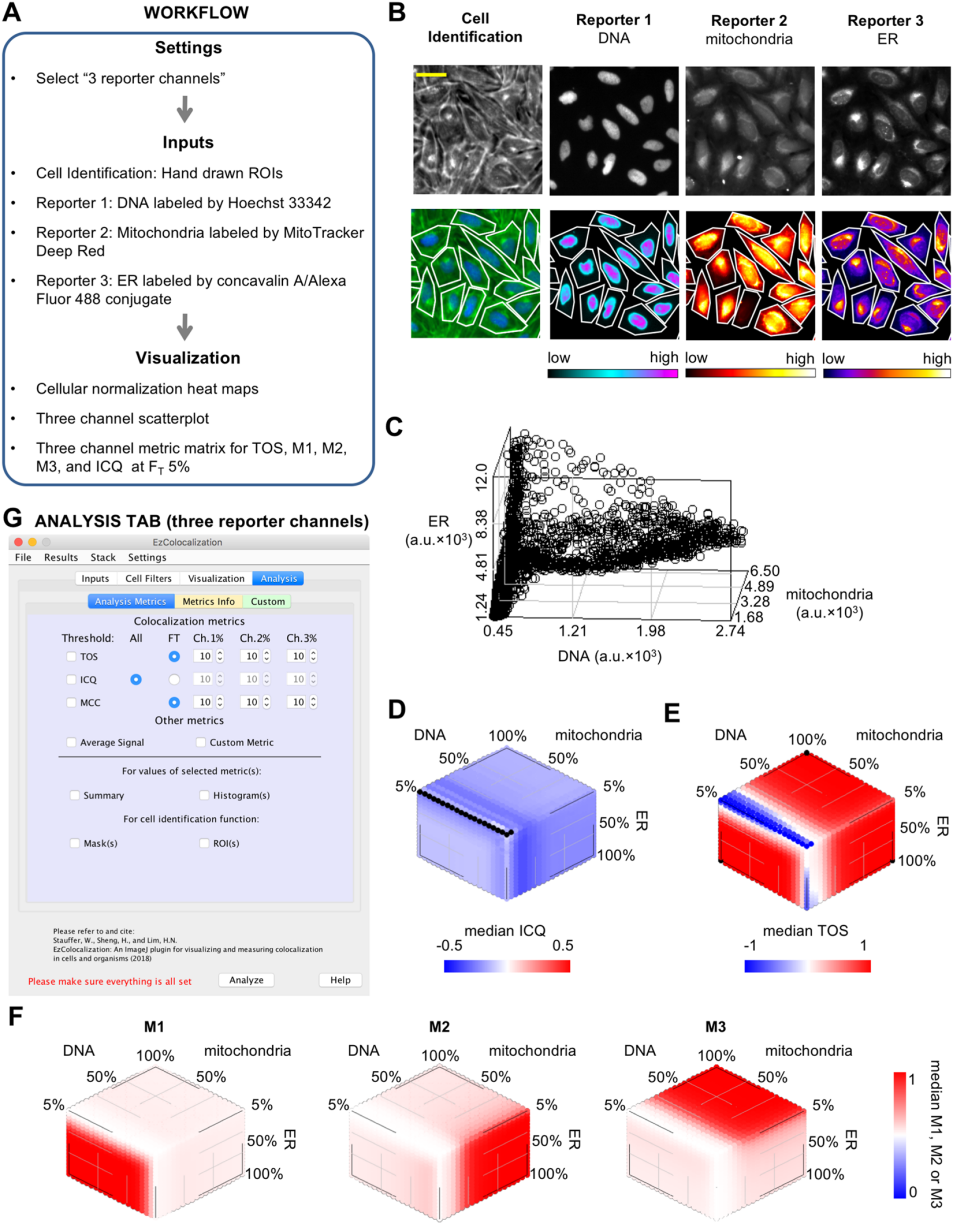
Application 4: Measurement of colocalization for three reporter channels. Images are of human bone cancer cells (U2OS) labelled as described in the main text. (**A**) Workflow of the analysis. (**B**) Images of cells in the cell identification and reporter channels. Top row are raw images. Bottom row, left image is the cell identification with pseudocolor (blue is the signal from Hoechst 33342 signal and green is the signal from phalloidin/Alexa Fluor 568 conjugate and wheat germ agglutinin/Alexa Fluor 555 conjugate) and boundaries of the ROIs in white (see main text). Bottom row (except left image) are heat maps for each of the three reporters with the boundaries of the ROIs shown. Signal intensity is indicated by the bar below each reporter image. Scale bar is 20 μm. (**C**) A three channel scatterplot for a single cell is shown for illustrative purposes only. (**D–F**) Metric matrices of median values for ICQ (**D**) TOS (**E**) and Manders’ colocalization coefficients M1, M2 and M3 (**F**) for all cells in the analysis (*n* = 66). Note: black color on metric matrix for ICQ indicates there were no pixels above all three thresholds for some cells, and therefore ICQ could not be calculated. (**G**) Analysis Metrics subtab for the Analysis tab for three reporter channels.

EzColocalization was tested on images from experiments and on modified images created to test specific issues (*e.g*. misalignment). Unpublished images of bacterial cells (HL6187) were used to illustrate the different modules of EzColocalization (Figs 1–4). These bacteria had plasmid pHL1392 in strain HL3338^23^. pHL1392 has the ampicillin resistance gene, ColE1 origin, and the green fluorescent protein (GFP) fused to part of the *sodB* gene and transcribed from the PLlacO-1 promoter. The sources of the images used for the application experiments (Figs 5–8) are stated in the relevant Results section. Note: images presented in the figures are cropped so that it is easier to see individual cells.

### Download and installation

For users without ImageJ, the first step is to download and install the ImageJ application from: https://imagej.nih.gov/ij/download.html. The next step is to download the EzColocalization plugin from: http://sites.imagej.net/EzColocalization/plugins/. When saving the file, the user should delete the timestamp at the end of the name of the EzColocalization file. For example, a version named “EzColocalization_.jar-20180716210728” should be renamed as “EzColocalization_.jar”. Once the plugin has been renamed “EzColocalization_.jar” it can be moved into the “plugins” folder of ImageJ to install it. Alternatively, users can install it by running ImageJ, selecting “Install…” from the “Plugins” menu of the menu bar, and then selecting the renamed file to install. To use EzColocalization, run the ImageJ application (open “ImageJ.exe” in the ImageJ folder) and choose “EzColocalization” from “Plugins” on the menu bar. For those using Fiji, the EzColocalization update site can be followed according to the instructions at https://imagej.net/Following_an_update_site.

## Results

### Overview of EzColocalization workflow

The workflow for EzColocalization is divided into four modules each with its own tab on the GUI. The tabs are: (i) “Inputs” where images, masks or regions of interest (ROI) lists are selected and aligned; (ii) “Cell Filters” where cells can be selected based on physical features and signal intensity; (iii) “Visualization” where heat maps, scatterplots, and metric matrices (defined below) are created; and (iv) “Analysis” where the colocalization metrics and outputs are chosen. Not all modules and not all processes within a module have to be used. Some tabs have a “Preview” button to run a specific module instead of the “Analyze” button which runs all selected processes in all modules.

### Inputs

Image files, which are chosen in the “Inputs” tab (Fig. 1A), must be: (i) monochromatic (*i.e*. not RGB or CMYK formats); (ii) 8-bit, 16-bit, or 32-bit; and (iii) in a format such as TIFF that retains the original pixel intensity values. Large images may be compressed for file transfer using a lossless format such as ZIP or LZW, and then decompressed for analyses. In addition to images, EzColocalization can accept masks and ROI lists for cell identification (see below). If there are multiple images for each channel, the images should be stacked for more efficient analysis in the “Stack” menu (see ImageJ guide for further details^24^). Images in a stack may be different fields of view or a time series, but must have the same dimensions, magnification and image order for each channel. The input tab also provides options for setting thresholds for signal intensity and aligning misaligned images from different channels (Fig. 1B and Supplementary Information). Recommendations for acquiring suitable images for colocalization analysis are provided in the Supplementary Information. Note: alignment operates on the assumption that an appropriate threshold for signal intensity can be chosen to distinguish pixels inside and outside of cells; if thresholding includes areas outside the cell or only a limited area within cells, then the alignment may not function properly. For this reason, all alignments should be checked by visually by examining the ROIs to confirm that appropriate cell areas are selected.

EzColocalization is primarily designed for one “cell identification” channel and two or three “reporter” channel images. However, it can operate with other input combinations (Table S1). The cell identification channel is used to identify individual cells, and consequently to distinguish intracellular and extracellular pixels. The cell identification channel can be any type of image that permits identification of the cell boundaries including: light microscopy images (*e.g*. phase contrast^25,26^ and bright-field), images with a reporter that labels the cell membrane or that is throughout the cytoplasm (*e.g*. Cy5, Fig. 1B), and images with an extracellular dye that outlines cells. Differential interference contrast (DIC) images create shadows that make it difficult for automated selection of cells using threshold methods^27^; therefore for DIC images we recommend that ROIs be created using the “selection tools” in ImageJ to manually outline cell areas, and then adding them to a list by choosing “Add to Manager” (in “Selection” submenu of the “Edit” menu). Once the ROIs for all cells of interest in an image are selected, a binary mask can be created using the “Clear Outside” and “Autothreshold” functions of ImageJ.

### Cell Filters

The “Cell Filters” tab is used to help select cells in images (Fig. 2A) and distinguish intracellular and extracellular pixels. Cells are identified by: (i) choosing one of the ImageJ threshold algorithms^24^, or manually selecting the thresholds (which is done by selecting “*Manual*” from a drop-down list in the Inputs tab and pressing the “Show threshold(s)” button), to identify regions corresponding to cells in the cell identification channel (Fig. 2B); (ii) using watershed segmentation to separate touching objects in the cell identification channel images (optional) (Fig. 2B); (iii) selecting objects from the cell identification channel images based on physical parameters (Fig. 2C) and signal intensity (Fig. 2D). EzColocalization will attempt to automatically detect whether input images have dark or light background using skewness. Assuming there are more pixels in the background than in the cells, an image with positive skewness indicates a dark background and negative skewness indicates a light background. Users can also manually select whether the input images have dark or light background in the “Parameters…” options of the “Settings” menu. Cells that are only partly within an image, and therefore could provide misleading values, are automatically removed from analyses.

EzColocalization has one optional “Pre-watershed filter” and eight optional post-watershed filters (with the option to select more). Watershed segmentation can aid the separation of dividing and touching cells^28^ but it can also divide large objects such as aggregates of extracellular material into smaller fragments that are the same size as cells. To avoid the latter, the Pre-watershed filter can be used to exclude objects with large areas from the analysis. The Preview button in the Cell Filters tab allows users to see which objects on the current image will be selected when the minimum and maximum bounds of all the filters are adjusted. There are two classes of parameters for the post-watershed cell filters (Table S2): (i) physical parameters based on measurements from the cell identification channel; and (ii) signal intensity parameters from the reporter channels. Physical parameters apply to all channels whereas signal intensity parameters apply only to the reporter channel for which they are selected (because reporters may have very different levels of signal). In addition to filtering based on predefined options in ImageJ, EzColocalization has filters for the “MeanBgndRatio” or “MedianBgndRatio”, which are calculated by dividing the mean or median signal intensity of pixels inside an object by the respective mean or median signal intensity of extracellular pixels.

### Visualization

The “Visualization” tab displays signals or metrics in cells as: (i) “heat maps”; (ii) scatterplots; and (iii) “metric matrices” (Fig. 3A).

Heat maps are pseudocolor images that show the relative magnitude of reporter signals (Fig. 3B). They are generated by normalization and rescaling so that the minimum and maximum pixel values are 0 and 255 respectively in each cell, image, or stack. There are eight options for coloring the heat maps, and the intensity values for each color are obtained from the “Show LUT” function (within the “Color” submenu of the “Image” menu in ImageJ). Cell heat maps are suited for determining where each reporter occurs with highest intensity in cells. Image heat maps can show if different cells within a field of view have substantially different intensities, which may indicate biological heterogeneity or unevenness in labeling. Stack heat maps can show if cells in different images have substantially different levels of signal intensity, which may indicate unevenness in labeling or measurements across a slide (*e.g*. due to photobleaching) or changes in signal over time (if the stack is a time series). Note: heat map appearances are affected by brightness and contrast settings.

Scatterplots show the relationship between the signal intensity for two or three reporter channels for individual cells and images (Fig. 3C). This relationship is important in choosing the appropriate colocalization metric (Supplementary Information). Scatterplots can also reveal heterogeneity in the localization patterns^8^, which may require removal of background pixels or separate analyses for different cell types.

Metric matrices provide an overview of localization patterns by showing the calculated values of a colocalization metric for many threshold combinations. Metric matrices for the threshold overlap score (TOS) have been shown to be useful for the analysis of localization patterns for two reporter channels^8,15^ (Fig. 3D). For completeness, EzColocalization has the option to calculate metric matrices for two reporter channels using five other metrics: threshold overlap score with logarithmic scaling^8^, Pearson correlation coefficient (PCC), Manders’ colocalization coefficients (M1 and M2), Spearman’s rank correlation coefficient (SRCC), and intensity correlation quotient (ICQ)^8,15^. Colocalization for three channels can also be measured using ICQ, Manders’ colocalization coefficients and TOS^29^ (Supplementary Information).

Thresholds for all metrics are measured as the top percentile (F_T_) of pixels for signal intensity^8,15^. For example, F_T_ = 0.1 is the 10% of pixels with the highest signal. For the metric matrices, F_T_ is also used to specify the step size for the threshold combinations. That is, F_T_ = 0.1 also selects thresholds for the 10%, 20%, …, and 100% of pixels with the highest signal. If F_T_ does not divide evenly into 100, then the remaining percent is the last step size. For metrics that do not need a threshold (*i.e*. PCC, SRCC, and ICQ) the values are calculated assuming that only the pixels above the thresholds exist. The metric matrix window has options for the results to be saved as text or image, for changing the F_T_ or type of metric, viewing individual cell metric values as a list, and calculating the mean, median or mode of the metric for each threshold combination. The “Proc” (processed) and “Raw” button determines whether the list of data displayed, copied, or saved with the “List”, “Copy”, or “Save…” buttons respectively is the average value for the sample for each threshold combination (*e.g*. median value) or all values for each cell in the sample for all threshold combinations.

### Analysis

The “Analysis” tab has three subtabs (“Analysis Metrics”, “Metrics Info” and “Custom”). The Analysis Metrics subtab has six metrics for measuring colocalization for two reporters (Fig. 4A) and three metrics for three reporters (see previous section). Users may choose a threshold or no threshold for PCC, SRCC and ICQ. TOS and Manders’ colocalization coefficients must have a threshold to be calculated. The Metrics Info subtab contains information and resources about the metrics used in the Analysis Metrics subtab (more details in the Supplementary Information). Thresholds can be selected using Costes’ method^30^ or manually. In the Custom subtab (see Supplementary Information for additional information), users can write their own code in Java^TM^ to analyze images (note: the example provided is for calculating PCC) (Fig. 4B). The “Compile” button tests the code and creates a temporary file in the Java temporary directory and displays the outcome of the compiling with a “Succeeded” or “Failed” label. If successful, the compiled custom code is read to the memory again and applied to the selected cells.

The output of every analysis is a table that specifies the image and an identifier number for every cell (Fig. 4C), and for each cell, values are provided for: (i) the selected metric; (ii) physical parameters; and (iii) average signal intensity for each channel (if selected). Note: “NaN” in the output table indicates the failure to calculate a value. Users can also generate summary windows (with the cell number, mean, median and standard deviation for the selected metric) (Fig. 4D), histograms of metric values (Fig. 4E), binary mask images, and a list of ROIs that represent each cell’s position and number on each image in the ROI manager. ROI lists and binary mask images can be saved for re-analysis of the same cells.

### Applications of EzColocalization

EzColocalization is designed to be used in a modular manner to facilitate customization of analyses for a wide variety of experiments and researcher needs. This section focuses on demonstrating specific tools in EzColocalization to solve real-world problems in diverse image sets.

In the first application of EzColocalization, images of rat hippocampal neurons from the Cell Image Library (CIL:8773, 8775–8788, which are attributed to Dieter Brandner and Ginger Withers) are used to demonstrate: (i) the use of a reporter channel for cell identification when an experiment does not have separate non-reporter images for cell identification; (ii) cell filters for selecting cells; and (iii) visualization tools for choosing metrics. The workflow of the analysis is outlined in Fig. 5A. In the first step, two reporter image stacks were created: one stack with images where F-actin is labelled (using a phalloidin peptide conjugated to rhodamine); and the second stack with images where tubulin is labelled (using an antibody conjugated to Alexa 488) (Fig. 5B). The interaction of F-actin and tubulin is important for the growth and migration of neurons^31,32^. We used the F-actin images for cell identification because it is present in all cells and it shows the cell boundaries^8^. Individual cells were selected from the F-actin images by applying a threshold to identify cells^24^ and using a cell filter to remove cell debris (note: parameter values in Fig. 5A).

After the cells were selected, the intensity of reporter signals were examined using cellular heat maps and scatterplots. We found the reporters did not colocalize at high signal levels and there was a complex relationship between the signal intensities (Fig. 5C,D). Due to the latter, localization was quantified using Manders’ M1 and M2 and TOS (Supplementary Information). M1 and M2 were evaluated at thresholds selected by Costes’ method for the cell outlined in Fig. 5B, and the values were 0.289 and 0.995 respectively. These values are usually interpreted as indicating that tubulin has high colocalization with F-actin, and F-actin has low colocalization with tubulin. TOS values were evaluated by generating a metric matrix with median TOS values. The matrix showed colocalization, anticolocalization and noncolocalization at different thresholds for the signal intensities of tubulin and F-actin (Fig. 5E). At sites in cells where F-actin and tubulin have the highest intensity signal (top 10% of pixels for each channel), the median TOS value is −0.36 (*n* = 20). This negative value indicates anticolocalization, which is consistent with the impression obtained from the heat maps and scatterplots, and with other reports^8^.

In the second application, images of *Saccharomyces cerevisiae* undergoing mitosis were obtained from the Cell Image Library^33^ to demonstrate: (i) cell identification via hand-drawn outlines (for experiments where automated methods of cell identification cannot be applied); and (ii) image alignment. The reporter inputs were an image from a wild type strain (“control”; CIL: 13871) that has the BFA1 protein that loads TEM1 onto the spindle pole body, and an image from a strain without the BFA1 protein (∆*bfa1* deletion mutant; CIL: 13870). In these reporter images, cells expressed TEM1 protein fused to GFP and the DNA was labelled with DAPI (4′, 6-diamidino-2-phenylindole). TEM1 localizes to spindle pole bodies during mitosis and is implicated in triggering exit from mitosis^33^. The workflow is shown in Fig. 6A. In this application, ROIs were manually drawn around cells using the “Freehand” selection tool in ImageJ on DIC images. Binary masks, which were used to select cell areas, were created by selecting the ROIs and using the “Clear Outside” and then “Auto Threshold” functions of ImageJ^24^ (Fig. 6B). The cell areas were used for cell identification and to correct alignment between the DIC images and the reporter channels using the “Default” threshold algorithm (Fig. 6C). Following this cell identification and image alignment, the images are now ready for visualization and analysis as described in the previous example.

In the third application, images of whole adult *Caenorhabditis elegans* obtained from the Broad Bioimage Benchmark Collection (BBBC012v1, M14)^34^ were used to demonstrate that: (i) EzColocalization can analyze colocalization in whole organisms; and (ii) “cell” filters can select individual organisms based on reporter signal intensity. The images in this example are from the same dataset used in our study describing TOS (but they are not the same images)^8^. The workflow is shown in Fig. 7A. Outlines of individual *C. elegans* were drawn in ImageJ on bright-field images to create ROIs, and the ROIs were added to the ROI manager for “cell” identification. GFP expressed from the *clec-60* promoter in the anterior intestine was reporter 1 and mCherry expressed from the *myo-2* promoter within the pharynx, which is an organ next to the anterior intestine^35^, was reporter 2. Cell filters for physical parameters were unnecessary because only those objects considered to be suitable *C. elegans* had outlines drawn around them in the first place. However, cell filters for signal intensity were necessary because some *C. elegans* had low GFP signal, possibly due to transgene silencing^36,37^ (Fig. 7B). Subsequent visualization and analysis can be performed as described in the first application.

In the fourth application, we demonstrate the analysis of colocalization for three reporter channels. The workflow was the same as for two reporter channels except “3 reporter channels” was first selected in the “Settings” main menu (Fig. 8A). Images were obtained from the Broad Bioimage Benchmark Collection (BBBC025, Version 1, Image set: 37983, image: p23_s9) of U2OS bone cancer cells (*n* = 66)^38^. The three reporter images had DNA, endoplasmic reticulum (ER) and mitochondria respectively stained with Hoechst 33342, concanavalin A/Alexa Fluor488 conjugate, and MitoTracker Deep Red (upper row, Fig. 8B). Cell identification was performed with an image of the plasma membrane labeled with wheat germ agglutinin (WGA)/Alexa Fluor 555 conjugate (upper left, Fig. 8B). Note: the image also had the Golgi apparatus and F-actin network labeled^38^. The plasma membrane was traced using the polygon selection tool in ImageJ to create ROIs for the individual cells, and the ROI manager containing the ROIs was selected for cell identification.

The localization patterns were visualized in the same manner as for two reporters except that: (i) there are three sets of heat maps for the reporters instead of two (lower row, Fig. 8B); and (ii) scatterplots and metric matrices are in three dimensions (Fig. 8C–F). There is the option in the Visualization tab and the Analysis tab (Fig. 8G) to measure colocalization for the three reporters using ICQ, TOS or Manders’ M1, M2 and M3 metrics. Of the three metrics, we found that TOS was the easiest to interpret. TOS has a single value for measuring the colocalization of all three reporter signals, and it clearly showed the reporter signals for the nucleus, mitochondria and ER overlapped at low thresholds (*i.e.* at high F_T_ values there is colocalization; red color in Fig. 8E) and did not overlap at high thresholds (*i.e.* at low F_T_ values there is anticolocalization; blue color in Fig. 8E). These observations are consistent with the nucleus, mitochondria and ER organelles overlapping at their edges (where the signal from their reporters is typically lower) due to known physical interactions, but not at their centers (where the signal from their reporters is typically higher) because they are distinct structures in cells^39–41^.

## Discussion

EzColocalization was designed to make it easier for researchers to determine where particular types of molecules occur in cells and organisms in relation to other types of molecules. In addition, EzColocalization can provide data on colocalization for each cell or organism in a sample, which is increasingly recognized as being crucial to understanding biological processes such as cell differentiation^42^, cancer^43^, and microbial pathogenesis^44^.

Two of the most widely used applications for colocalization analysis are JACoP and Coloc2^10,12^. JACoP is an ImageJ plugin that can generate pixel intensity scatterplots to visualize localization patterns and measure colocalization with a variety of metrics including PCC (Van Steensel’s CCF method or Costes’ randomization), Manders’ M1 and M2, ICQ, and object based methods^10^. It also permits thresholds to be chosen manually or automatically using Costes’ method^10^. Coloc 2 is a plugin for Fiji^12^, which builds on the functionality of JACoP by providing options to: analyze selected ROIs within single images, threshold images using a “bisection” algorithm, and measure colocalization with SRCC and Kendall’s Tau rank correlation. Unfortunately, JACoP and Coloc 2 do not have built-in options to automate analyses or perform separate colocalization measurements for multiple objects in an image, therefore analyses can be challenging for images with a lot of background pixels or different cell types. The Wright Cell Imaging Facility (WCIF) has helped address these challenges by creating a colocalization plugin that can measure colocalization for individual cells by manually creating individual ROIs^11^, but this method cannot be easily automated to analyze many cells across many images.

In addition to the above, software has been reported for measuring colocalization in cells, particularly in cases where the signal is defined to distinct regions or foci. One of these applications is MatCol, which can identify overlapping objects after a threshold is applied, and then calculate if the measured overlap is significantly different to that expected if the same objects were randomly scattered^45^. Another reported script calculates object based colocalization in confocal images^46^ from the percent overlap of the objects. A third program measures colocalization for three-dimensional images; it measures the proportion of thresholded objects in one channel that have their center of mass within thresholded objects of another channel^47^. There are practical barriers to the widespread use of these three programs including the need for additional software to identify cell areas and that they are written in Matlab or C++ (therefore users must be familiar with these programming languages to customize them).

To make it easy to optimize analyses, EzColocalization has a simple GUI that requires no programming experience unless a custom metric is created. The GUI template is based on one that is familiar to many microscopists. ImageJ also has a large library of tools that can be used with EzColocalization, and it is open source software^24^. ImageJ has options for creating stacks of images and thresholding images, which were incorporated into EzColocalization for automated analyses. EzColocalization also has tools for the input of images, cell identification, visualizing localization patterns, measuring colocalization, and for displaying and saving results.

EzColocalization can select individual cells from cell identification images using thresholds, ROIs, or binary mask images. Identification of individual cells allows pixels within cells to be discriminated from pixels in the background and non-cell objects. In addition, cell filters can limit analyses to a subset of cells with certain physical parameters and minimum signal levels. Filters are used to select cells instead of more advanced techniques for cell detection^48^ because: (i) they do not require assumptions about cell features (therefore diverse cell types can be analyzed); and (ii) they are intuitive, which makes it easier for researchers to tailor settings for their experiments and identify if patterns of localization are associated with specific cell features.

The visualization tools (heat maps, scatterplots, and metric matrices) can help with choosing the appropriate metrics and thresholds for the analyses. The metric matrices are particularly useful for samples with non-specific binding or localization of probes. These matrices display colocalization values for multiple combinations of thresholds for signal intensity, which facilitates the selection of thresholds so the analysis includes pixels from cellular regions with high signal (due to specific localization) and excludes pixels from regions with low signal (due to non-specific localization).

EzColocalization can not only measure colocalization for two reporters but also for three reporters. The latter is a useful feature that is unavailable for most software applications for measuring colocalization. In addition, custom metrics can be programmed in EzColocalization.

The data table generated by the colocalization analysis is an important feature of EzColocalization. Because the value of the colocalization metric for each cell is provided, and not just the average measurement of colocalization for the sample, it is possible to examine the distribution of metric values, perform statistical analyses, calculate receiver operating characteristic curves, and analyze subsets of cells in heterogeneous samples^8^. The data table also lists the specific image and a unique identifying number for each cell, therefore researchers can examine the images to determine why different cells have different measurements. The data tables can be downloaded and used in any spreadsheet application, which makes the data accessible to researchers without programming experience. Furthermore, the values for the physical parameters, signal intensity, and colocalization metrics can be retrieved from the tables (if the check box is selected) for more sophisticated multivariate analyses, including clustering, classifying and ordination methods.

In conclusion, EzColocalization is an ImageJ plugin with a user-friendly GUI, tools for start-to-finish analysis of colocalization, and many options to customize analyses. The tools are provided to select specific types of cells or organisms, visualize and measure colocalization, and automate analyses. The analysis generates a data table with measurements of colocalization, signal intensity and physical parameters for each cell, which allow users to delve deep into their data. Together these features make EzColocalization ideal for researchers at all levels, and for analyzing heterogeneous samples and complex patterns of localization.

## Electronic supplementary material

Supplementary Information

## References

[1] Kannaiah S, Amster-Choder O (2015). Methods for studying RNA localization in bacteria. Methods.

[2] Kocaoglu O, Carlson EE (2016). Progress and prospects for small-molecule probes of bacterial imaging. Nat Chem Biol.

[3] Xue L, Karpenko IA, Hiblot J, Johnsson K (2015). Imaging and manipulating proteins in live cells through covalent labeling. Nat Chem Biol.

[4] Gautam S, Gniadek TJ, Kim T, Spiegel DA (2013). Exterior design: strategies for redecorating the bacterial surface with small molecules. Trends Biotechnol.

[5] Gruskos JJ, Zhang G, Buccella D (2016). Visualizing Compartmentalized Cellular Mg2+ on Demand with Small-Molecule Fluorescent Sensors. J Am Chem Soc.

[6] Perry JL, Ramachandran NK, Utama B, Hyser JM (2015). Use of genetically-encoded calcium indicators for live cell calcium imaging and localization in virus-infected cells. Methods.

[7] Kervrann C, Sorzano CÓS, Acton ST, Olivo-Marin JC, Unser M (2016). A guided tour of selected image processing and analysis methods for fluorescence and electron microscopy. IEEE Journal of Selected Topics in Signal Processing.

[8] Sheng H, Stauffer W, Lim HN (2016). Systematic and general method for quantifying localization in microscopy images. Biol Open.

[9] Adler J, Parmryd I (2014). Quantifying colocalization: thresholding, void voxels and the H(coef). PLoS One.

[10] Bolte S, Cordelieres FP (2006). A guided tour into subcellular colocalization analysis in light microscopy. J Microsc.

[11] Dunn KW, Kamocka MM, McDonald JH (2011). A practical guide to evaluating colocalization in biological microscopy. Am J Physiol Cell Physiol.

[12] Schindelin J (2012). Fiji: an open-source platform for biological-image analysis. Nat Methods.

[13] Yao Z, Carballido-López R (2014). Fluorescence imaging for bacterial cell biology: from localization to dynamics, from ensembles to single molecules. Annual review of microbiology.

[14] Haas BL, Matson JS, DiRita VJ, Biteen JS (2014). Imaging live cells at the nanometer-scale with single-molecule microscopy: obstacles and achievements in experiment optimization for microbiology. Molecules.

[15] Sheng H, Stauffer WT, Hussein R, Lin C, Lim HN (2017). Nucleoid and cytoplasmic localization of small RNAs in Escherichia coli. Nucleic Acids Res..

[16] Snapp Erik (2005). Design and Use of Fluorescent Fusion Proteins in Cell Biology. Current Protocols in Cell Biology.

[17] Wallner G, Amann R, Beisker W (1993). Optimizing fluorescent *in situ* hybridization with rRNA-targeted oligonucleotide probes for flow cytometric identification of microorganisms. Cytometry.

[18] Patterson GH, Knobel SM, Sharif WD, Kain SR, Piston DW (1997). Use of the green fluorescent protein and its mutants in quantitative fluorescence microscopy. Biophys J.

[19] Li B, You L (2013). Predictive power of cell-to-cell variability. Quantitative Biology.

[20] Wiegand WE (2004). A platform for integrating development tools. IBM Systems Journal.

[21] Réveillac, J.-M. *Modeling and Simulation of Logistics Flows 2: Dashboards, Traffic Planning and Management*. (John Wiley & Sons, Inc., 2017).

[22] Clayberg, E. & Rubel, D. *Eclipse Plug-ins*. Third edn, (Addison-Wesley, 2008).

[23] Hussein R, Lim HN (2011). Disruption of small RNA signaling caused by competition for Hfq. Proc Natl Acad Sci USA.

[24] Ferreira, T. & Rasband, W. *ImageJ User Guide — IJ 1.46*. https://imagej.nih.gov/ij/docs/guide/user-guide.pdf (2012).

[25] Zernike F (1942). Phase contrast, a new method for the microscopic observation of transparent objects. Physica.

[26] Zernike F (1942). Phase contrast, a new method for the microscopic observation of transparent objects part II. Physica.

[27] Obara B, Roberts MA, Armitage JP, Grau V (2013). Bacterial cell identification in differential interference contrast microscopy images. BMC Bioinformatics.

[28] Vincent L, Soille P (1991). Watersheds in Digital Spaces - an Efficient Algorithm Based on Immersion Simulations. Ieee Transactions on Pattern Analysis and Machine Intelligence.

[29] Manders EMM, Verbeek FJ, Aten JA (1993). Measurement of colocalization of objects in dual-colour confocal images. J Microsc.

[30] Costes SV (2004). Automatic and quantitative measurement of protein-protein colocalization in live cells. Biophys J.

[31] Rodriguez OC (2003). Conserved microtubule-actin interactions in cell movement and morphogenesis. Nat Cell Biol.

[32] Coles CH, Bradke F (2015). Coordinating neuronal actin-microtubule dynamics. Curr Biol.

[33] Valerio-Santiago M, Monje-Casas F (2011). Tem1 localization to the spindle pole bodies is essential for mitotic exit and impairs spindle checkpoint function. J Cell Biol.

[34] Ljosa V, Sokolnicki KL, Carpenter AE (2012). Annotated high-throughput microscopy image sets for validation. Nat Methods.

[35] Wahlby C (2012). An image analysis toolbox for high-throughput C. elegans assays. Nat Methods.

[36] Kelly WG, Xu S, Montgomery MK, Fire A (1997). Distinct requirements for somatic and germline expression of a generally expressed Caernorhabditis elegans gene. Genetics.

[37] Hsieh J (1999). The RING finger/B-box factor TAM-1 and a retinoblastoma-like protein LIN-35 modulate context-dependent gene silencing in Caenorhabditis elegans. Genes Dev.

[38] Bray M-A (2016). Cell Painting, a high-content image-based assay for morphological profiling using multiplexed fluorescent dyes. Nature protocols.

[39] English AR, Voeltz GK (2013). Endoplasmic Reticulum Structure and Interconnections with Other Organelles. Cold Spring Harbor Perspectives in Biology.

[40] Marchi S, Patergnani S, Pinton P (2014). The endoplasmic reticulum–mitochondria connection: One touch, multiple functions. Biochimica et Biophysica Acta (BBA) - Bioenergetics.

[41] Prachar J (2003). Intimate contacts of mitochondria with nuclear envelope as a potential energy gateway for nucleo-cytoplasmic mRNA transport. General physiology and biophysics.

[42] Potente M, Makinen T (2017). Vascular heterogeneity and specialization in development and disease. Nat Rev Mol Cell Biol.

[43] Meacham CE, Morrison SJ (2013). Tumour heterogeneity and cancer cell plasticity. Nature.

[44] Ackermann M (2015). A functional perspective on phenotypic heterogeneity in microorganisms. Nat Rev Microbiol.

[45] Khushi M, Napier CE, Smyth CM, Reddel RR, Arthur JW (2017). MatCol: a tool to measure fluorescence signal colocalisation in biological systems. Sci Rep.

[46] Kreft M, Milisav I, Potokar M, Zorec R (2004). Automated high through-put colocalization analysis of multichannel confocal images. Comput Methods Programs Biomed.

[47] Fletcher PA, Scriven DR, Schulson MN, Moore ED (2010). Multi-image colocalization and its statistical significance. Biophys J.

[48] Buggenthin F (2013). An automatic method for robust and fast cell detection in bright field images from high-throughput microscopy. BMC Bioinformatics.

