# Supplementary material for: EzColocalization: An ImageJ plugin for visualizing and measuring colocalization in cells and organisms
Source: Sci Rep. 2018 Oct 25;8:15764. doi: 10.1038/s41598-018-33592-8 (PMC6202351; doi:10.1038/s41598-018-33592-8)
Supplement: Supplementary file 1 — Supplementary Information [file 41598_2018_33592_MOESM1_ESM.pdf]

SUPPLEMENTARY INFORMATION

EzColocalization: An ImageJ plugin for visualizing and measuring colocalization in cells and organisms

Weston Stauffer, Huanjie Sheng and Han N. Lim

Department of Integrative Biology, University of California Berkeley

Contents

1. Description of packages and classes for EzColocalization

2. Data acquisition guidelines

3. Image alignment

4. Heat maps, scatterplots and metric matrices

5. Colocalization metrics for two reporter channels

6. Colocalization metrics for three reporter channels

7. Custom analysis

8. Supplementary references

9. Supplementary figure legends

1. Description of packages and classes for EzColocalization

The first two packages have very basic purposes. The first is the “default” package (by Java™ convention) and its only function is to load the plugin within ImageJ. This package contains a single class, “EzColocalization_”, and outputs from this package are not accessible by other classes in other packages. The second package is “ezcol.files”, which has a single class (“FilesIO”) that loads all emblems and sample images for the GUI.

The third package is “ezcol.main”. It performs shared and general functions, and has six classes (“GUI”, “ImageInfo”, “MacroHandler”, “PluginStatic”, “PluginConstants”, and “AnalysisOperator”). GUI creates the GUI. ImageInfo stores information on the formats of the input images. MacroHandler enables use of the recorder in ImageJ so users can run macros that automatically run commands in batches. For example, the recorder can be used to create a macro to automatically modify and analyze a large set of images with particular settings. PluginStatic contains all static parameters (inputs) and static utility methods (common functions) used in analyses. PluginConstants contains all shared constants that are accessed by other classes. AnalysisOperator coordinates the operation of analyses in response to the inputs.

The fourth package is “ezcol.align”, which performs image alignment and has three classes (“BackgroundProcessor”, “TurboRegMod”, and “ImageAligner”). BackgroundProcessor enhances the contrast of images by: (i) subtracting background signal from pixels using the rolling ball algorithm in the “Subtract Background” function of ImageJ [1]; (ii) generating binary images of the reporter and cell identification channels with a user chosen algorithm from the “Auto Threshold” function in ImageJ [1] or thresholds manually set by the user (note: only a single manual threshold can be applied for a stack of images, and this is performed by selecting “*Manual*” and then displaying the thresholds by selecting “Show threshold(s)”; if no manual selection is made, the “Default” algorithm is applied); (iii) converting all pixels above the value identified by the Auto Threshold algorithm to a value of 255, and all those below it to 0; (iv) applying the “Fill Holes” function of ImageJ [1] on the binary images to better select the entire area of cells; and (v) calculating the average signal of pixels below the threshold in each reporter channel.

TurboRegMod uses the “Translation” alignment algorithm from TurboReg [2] to calculate the required XY coordinate shifts to align the binary images from the output of the BackgroundProcessor class by maximizing the overlap of pixels above the threshold. Note: interpolation of pixel values and other alignment functions that are normally performed by TurboReg are avoided because these functions alter pixel values. ImageAligner performs the image alignment by applying the calculated XY shifts from TurboRegMod to the original images.

The fifth package is “ezcol.cell”, which identifies cell areas and obtains pixel values. This package has six classes (“ParticleAnalyzerMT”, “CellFinder”, “CellFilterDialog”, “CellDataProcessor”, “DataSorter”, and “CellData”). Note: “cell” refers to any objects being analyzed, including subcellular structures or whole organisms. ParticleAnalyzerMT is a customized multithreading version of the “Analyze Particle” function from ImageJ [1] and it is used to identify cell areas above the thresholds, which are pixels of the objects on the binary images produced by BackgroundProcessor (see previous package)). CellFinder takes inputs from the previous class and converts them into a format for the next class, performs watershed segmentation [1], and removes cells based on user defined cell filters. CellFilterDialog opens the window for additional cell filters. CellDataProcessor obtains the values of pixels identified for each cell. DataSorter and CellData sort the pixel values of cells based on intensity and store them so that these steps do not need to be repeated multiple times for later calculations.

The sixth package is “ezcol.metric”, which performs colocalization analysis in response to inputs from ezcol.cell, and contains six classes (“BasicCalculator”, “MetricCalculator”, “CostesThreshold”, “MatrixCalculator”, “MatrixCalculator3D”, and “StringCompiler”). BasicCalculator is an abstract class containing methods and values shared by the other “calculators” (*i.e.* MetricCalculator, MatrixCalculator, MatrixCalculator3D). MetricCalculator uses previously described algorithms to calculate Li’s ICQ [3], Manders’ colocalization coefficients M1, M2 [4] and M3, PCC [5], SRCC [6], and TOS [7]. “CostesThreshold” uses Costes’ method for determining a threshold [8] and the algorithm was optimized for faster operation using ranked pixel values and dynamic programming as follows. The thresholds start at the maximum pixel values for each channel and PCC is calculated. Then the thresholds are decreased to the next

highest pixel value, the values above the new threshold are subtracted from the stored sums, and PCC is calculated again from the new stored sums, and so on. During the entire process when all the pixels have been removed, we compare all the PCC values calculated for all thresholds to find the minimum absolute PCC value. MatrixCalculator calculates metric matrices for two reporter channels. MatrixCalculator3D creates metric matrices for three reporter channels. StringCompiler compiles and performs any custom analysis written by the user.

The seventh and eighth packages are “ezcol.visual.visual2D” and “ezcol.visual.visual3D”, which output plots and data from the analyses. These packages are located in the folder called “visual” and both obtain inputs from ezcol.cell for heat maps and scatterplots and from ezcol.metric for histograms and metric matrices.

The ezcol.visual.visual2D package contains nine classes for visualizing two dimensional data and results (“HeatGenerator”, “HeatChart”, “HistogramGenerator”, “HistogramStackWindow”, “ScatterPlotGenerator”, “PlotStackWindow”, “HeatChartStackWindow”, “OutputWindow”, and “ProgressGlassPane”). HeatGenerator normalizes pixels values so the maximum and minimum values are 0 and 255 (8-bit) or 65535 (16-bit) respectively for each cell, image, or stack. The normalized values are assigned colors from ImageJ lookup tables [9], or assigned from Matlab (R2015a, Mathworks, Natick, MA, USA) in the case of “hot” and “cool” colors. HeatChart is a modified version of the class JHeatChart (created by Tom Castle) which takes colors from the previous class to generate heat maps as RGB images, and values from MatrixCalculator to generate two dimensional metric matrices. HistogramGenerator and HistogramStackWindow generate histograms by respectively converting cell based data into histogram data starting with ten bins, and generating a stack of histograms for selected metrics. The number of bins can be increased or decreased in increments of one with the “nBin+” or “nBin−” buttons. ScatterPlotGenerator obtains pixel values from two reporter channels for five random cells per image in a stack. If five or less cells are present in an image, then pixel values are obtained for all cells in the image. PlotStackWindow creates and displays a stack of scatterplots, with each plot containing the pixel values for a single cell. HeatChartStackWindow generates the metric matrices window. OutputWindow generates

the analysis summary window and its content. ProgressGlassPane generates the progress bar and presents tips in the GUI.

The ezcol.visual.visual3D package has 14 classes for visualization of three reporter channels in dynamic three dimensional scatterplots and metric matrices (“Arrow3D”, “Cube3D”, “Element3D”, “GraphicsB3D”, “Line3D”, “Point3D”, “Polygon3D”, “Rect3D”, “Renderer”, “ScatterPlot3D”, “ScatterPlot3DWindow”, “Spot3D”, “Square3D”, and “Text3D”). All classes are adopted from the jaytools.jar written by Urah Jay. His original classes are modified particularly for three dimensional scatterplots. Element3D is an abstract class (which means it cannot be initialized or constructed) containing methods and values shared by the other classes, including “Arrow3D”, “Cube3D”, “Element3D”, “Line3D”, “Point3D”, “Polygon3D”, “Rect3D”, “Spot3D”, “Square3D”, and “Text3D”. These classes represent the corresponding 3D elements as their names suggest; for example, “Arrow3D” is a class to indicate an arrow on a 3D graph. Some of these 3D elements (“Arrow3D”, “Polygon3D”, “Rect3D”, and “Square3D”) are not used for the purpose of this plugin but are kept for completeness of the package. Renderer is the 3D graphics process of automatically converting 3D elements into 2D image data, the results of which are feed into GraphicsB3D to paint the 2D image data of the projected 3D elements on the image canvas. ScatterPlot3D and ScatterPlot3DWindow generate 3D scatterplots and metric matrices by respectively converting the cell data into a compatible format for plotting and presenting the data in the plot window. 3D scatterplots are created in the same manner as ScatterPlotGenerator except for obtaining pixel values from three reporter channels. Usually the first cell is shown when the image window opens, and users can select the next cell or the previous cell by clicking the forward and back buttons on the window. 3D metric matrices are generated in the same way as HeatChart but all color squares are replaced by 3D spheres to enable visualization of deeper layers in the 3D matrices.

The ninth and final package is “ezcol.debug”. It has two classes, and reports errors and warnings within the plugin. It contains a class, “ExceptionReporter”, which handles and reports errors or warnings, and the class, “Debugger”, which was used during development to debug the plugin.

2. Data acquisition guidelines

Accurate colocalization measurements begin with good experimental data, which depends on the samples, the reporters, the imaging system, data collection methods, controls and replicate measurements. Some guidelines the authors have found useful include the following. Samples should be prepared in a manner that: preserves the native spatial organization, minimizes touching of cells or organisms (which makes the identification of individual cells or organisms easier), and minimizes movement of cells or organisms (especially for live imaging). The reporters should be optimized to specifically label the molecules of interest (which includes minimizing excess or non-specifically bound reporter), and to minimize cross-talk (also known as bleed-through) between the reporter signals and between each reporter signal and non-reporter signals in cells and tissues (*e.g.* autofluorescence). In addition, reporters with the highest specific signal should be preferentially paired with targets that have the lowest concentration. Too much reporter is sometimes more problematic for colocalization measurements than too little because of non-specific labeling or aggregation. A reporter that identifies the cell boundary or entire cell should be considered if the cell boundaries are unclear in bright-field imaging to facilitate automated cell identification so that single cell measurements of colocalization can be easily performed. The imaging system should be set-up with: a high quality monochromatic camera to maximize the signal-to-noise ratio, controls to check the settings and reproducibility of measurements on different days, and adjustments to the light source or neutral density filters to prevent oversaturated pixels with artificially low intensity values. It is important to recognize that misalignment between imaging channels often occurs (and may occur after the initial set-up and alignment) therefore images from different channels should ideally be overlaid in each experiment to evaluate the alignment and to correct any misalignment by adjusting the physical apparatus or the analysis. Differential interference contrast (DIC) is not recommended, and users should instead use phase contrast or another method that does not create shadows for identifying cell boundaries. Generally, it is preferable to maximize the resolution, but the scale of the cells and structures must be considered. For example, measuring the colocalization of reporters in intracellular structures will require a higher level of resolution than measuring colocalization at different tissue structures or organs. Additional guidance on the practical aspects of setting up a system for colocalization measurements is available in several reviews [10-12]. The data should be collected at the

highest number of bits to maximize the dynamic range of the signal, and images saved in an appropriate format (see note below). The importance of controls for the proper analysis of the colocalization measurements cannot be overstated. Researchers should not only include appropriate biological controls (*e.g.* deletions strains without the labeled protein) but should also measure some cells with only one of each reporter to quantify and to correct for bleed-through. In addition, independent replicate measurements of controls and samples on different days are important because labeling, microscopy set-up (especially in shared facilities), and any automated settings for image collection can vary dramatically between different experiments and often without the researcher being aware of it until the analysis is performed. As an aside, researchers should only use deconvolution or image corrections that have been proven to provide more accurate representation of localization for their specific reporters, samples, and imaging system.

The format of the images is important. The image file format should be TIFF or another lossless compression format with a single value for pixel intensity. A color camera that records pixel values in RGB can be problematic because it is unknown how the three values contribute to total signal intensity. Pseudocolors can be created for visualization purposes if the pixel intensity values are not changed. RGB and pseudocolor images can be distinguished by looking at the information on top of the image window in ImageJ.

3. Image alignment

The Inputs tab provides the option for the alignment of images from different channels. The alignment is performed by: (i) subtracting background signal from the cell identification and reporter images to enhance contrast using the rolling ball algorithm of the “Subtract Background” function (note: this step can be turned on or off in the “Parameters…” options of the “Settings” menu); (ii) thresholding the resulting images; (iii) creating a binary mask from the thresholded images; (iv) processing the binary mask with the “Fill Holes” function to ensure cell interiors are selected; (v) aligning the reporter channels and binary mask image using the translation alignment algorithm component of the TurboReg plugin [2]; (vi) obtaining the X and Y coordinate offset values from the alignment and using them to align the original cell identification and reporter images; and (vii) removing overhanging pixels and filling-in pixels (with a value of zero) so all images in the stacks have same size (yellow area in **Fig. 1B**). Note: TurboReg functions that interpolate pixel values are not used because they change the original values.

4. Heat maps, scatterplots and metric matrices

Many factors should be considered when performing analyses and selecting a metric for quantifying localization. These factors should include heterogeneity in the data, the specificity of the reporter, the relative intensity of the intracellular and extracellular background signals, and the relationship between the intensities of the reporter signals. EzColocalization provides tools in the Visualization tab to help users evaluate these considerations.

Heat maps created by EzColocalization can be normalized for each cell, each image, or each stack ("cell heat maps", "image heat maps" and "stack heat maps" respectively). Cell heat maps can help visually identify the locations in cells where reporters have the highest and lowest intensity, and the localization patterns (*i.e.* colocalization, anticolocalization and noncolocalization of the reporters). Image heat maps can show whether different cells have different average signal intensities within each image. The cell and image heat maps should be carefully inspected for evidence of heterogeneity among cells with respect to: the locations of reporters within cells, the localization pattern (*i.e.* relative positions of the reporters), and the average signal intensity. If there is heterogeneity, then it may be appropriate to limit analysis to a subpopulation of cells by using the cell filters in EzColocalization so that measurements are not an average of multiple populations. Image heat maps should also be examined to determine if the pixels with the highest signal (likely containing reporter) have similar levels of intensity to the pixels with the lowest signal ("background"). If so, then analysis may be improved by selecting individual cells from the image so that the only intracellular pixels are analyzed or by selecting thresholds so that only pixels with signal greater than background levels are analyzed (see metric matrices below) [6,10] .

Scatterplots reveal the relationship between the signal intensities for different reporters. Evaluating this relationship is important because different assumptions about the relationship of the reporter signals are central to the calculation, interpretation and selection of the metrics for colocalization (see next section). Scatterplots may also reveal if different cells or organisms within a sample have very different intensities or different relationships between pixel intensity. If there is heterogeneity, cell filters may be able to limit analysis to a more homogeneous population. In addition to cell-to-cell heterogeneity, there may be

heterogeneity within each cell; that is, different relationships between the signals at different levels of signal intensity. For example, a cell may have pixels with low signal for two reporters that have no correlation and pixels with high signal for the same two reporters that have a positive correlation (due to specific binding to a protein) [11]. In cases where there are different relationships between the pixels at different levels of signal, it may be possible to select thresholds for the reporter signals so that colocalization is only measured for a subset of pixels.

Metric matrices can be calculated for six different colocalization metrics in EzColocalization: TOS with linear or logarithmic scaling [7], PCC [12], SRCC [13], Manders' colocalization coefficients M1 and M2 [4,12] and ICQ [3]. Each metric matrix calculates the value of the selected metric at every combination of the thresholds chosen (**Fig. S1**). Metric matrices can quickly determine whether there are general patterns of colocalization, anticolocalization or noncolocalization that depend on signal intensity [7,14]. A metric matrix can also help to select a threshold that provides a better measure of colocalization for a subset of pixels with different intensities in a cell. That is, the selection of thresholds via the metric matrices can provide more targeted analysis. Because the thresholds in the metric matrix are measured in terms of the percentage of pixels rather than absolute signal level, the metric matrix is well-suited to comparing and aggregating values in a groups of cells where there may be some differences in average signal intensity and cell size.

In relation to selecting thresholds, EzColocalization provides two options: Costes' method and manual selection. Costes' method chooses the thresholds automatically [8]. The advantage of automatic selection of the thresholds is that it decreases the potential for user bias. However, the method often does not work well if the signal intensities of the intracellular and background pixels are not clearly distinguishable, the reporter signals do not have similar levels of intensity or a monotonic relationship, or there are outlier pixels with high signal [12]. Further, changing the order of reporter channels can result in different Costes' thresholds due to asymmetry in the linear regression used by the Costes' algorithm. Manual selection of thresholds by the user is more flexible but it requires care to ensure they are chosen appropriately. The heat map and scatterplots, as well as the metric matrices, can guide the manual selection of the

thresholds. Metric matrices can help ensure the thresholds are chosen so that they are representative of broad trends and the results are robust (*i.e*. a small change in the values of the thresholds should not substantially alter the result). Two notes of caution in regard to the selection of thresholds: (i) the metric matrix should not be used to “fish” for a metric and threshold values to give a result that is not broadly consistent with all the data; and (ii) the selection of thresholds must balance the need to eliminate pixels with background or non-specific signal against the need to keep as many pixels as possible so the results of the analysis are broadly representative and not fluctuating due to the noise associated with having a small number of values. Additional guidance on the selection of thresholds is provided in previous publications [7,14].

5. Colocalization metrics for two reporter channels

This section provides brief and general guidelines for selecting a colocalization metric. More detailed information on colocalization metrics is published elsewhere [7,10,11,15]. As mentioned in the previous section, it is important to examine the scatterplots to determine the relationship between the signal intensities of the reporters before choosing a colocalization metric.

Pearson's correlation coefficient (PCC) is the covariance of two variables divided by the product of their standard deviations. It is typically used to measure the linear correlation of the signal intensity values for two reporters (**Fig. S2**) [5,16]. PCC values can range from −1 which indicates a strong negative correlation between the signals (anticolocalization) to 1 which indicates a strong positive correlation (colocalization). A PCC value of 0 indicates there is no correlation (noncolocalization). Note: PCC could be used to measure nonlinear relationships following a nonlinear transformation of the data, although this is not typically done for measuring colocalization.

Spearman's rank correlation coefficient (SRCC) is calculated by ranking the pixels according to the intensity of signal for each channel and then measuring the correlation in the rankings between two channels [13]. SRCC measures whether the signal intensities of the reporters have a monotonic relationship (**Fig. S2**), and it is relatively insensitive to outliers because it is based on rankings. Therefore SRCC is suitable for non-linear, monotonic relationships such as power law or logarithmic functions. SRCC values range from −1 (anticolocalization) to 1 (colocalization) [15], and 0 indicates there is no correlation (noncolocalization).

The intensity correlation quotient (ICQ) is the ratio of the total number of pixels where the signal intensity is above the means for both channels or below the means for both channels (*i.e.* excluding pixels that are above the mean in one channel and below the mean in the second channel), divided by the total number of pixels, minus 0.5 [3,6]. ICQ ranges from –0.5 to +0.5. ICQ is essentially a sign test with positive or negative values for pixels that are on a positive or negative slope of a function through the mean of both channels. ICQ, like SRCC, is often used to evaluate whether the signal intensities of two reporters have a

monotonic relationship (although it could also be used for some types of non-monotonic relationships) (**Fig. S2**). ICQ is less sensitive to outliers than PCC. ICQ is not an appropriate metric for heterogeneous samples because the mean may not be an appropriate point around which localization should be evaluated.

Manders' colocalization coefficients M1 and M2 are calculated by determining the sum of the intensities of pixels that exceed thresholds for both signals 1 and 2 divided by the sum of the intensities of the pixels that exceed the threshold for signal 1 or by the sum of the intensities of the pixels that exceed the threshold for signal 2, respectively [4,12]. The threshold can be determined by several algorithms including Costes' threshold [8]. Disadvantages of M1 and M2 are that both values are needed to determine whether there is colocalization, and the interpretation of these values is complicated by them being dependent on the threshold values [17]. Manders' colocalization coefficients M1 and M2 (and also the threshold overlap score defined below) tend to be better for evaluating colocalization or anticolocalization in cases where there is not a clear localization pattern, there is a mixed pattern of localization, or there is a non-monotonic relationship (**Fig. S2**).

The threshold overlap score (TOS) is a newer metric that shares some similarity to M1 and M2 in that it calculates the overlap in pixels above a threshold [7,14]. TOS is calculated by determining the number of pixels that exceed thresholds for both signals 1 <u>and</u> 2 and dividing this number by the number of pixels that exceed the threshold for signal 1 <u>or</u> by the number of pixels that exceed the threshold for signal 2 (note: following normalization the same TOS value will be obtained using either denominator [7]). Unlike M1 and M2 there is no weighting for signal intensity. In addition, TOS divides the observed overlap by the overlap expected to occur simply by chance (which is not done for M1 and M2). A result of this normalization is that TOS measures colocalization as a single value which makes it easier to interpret and compare between experiments than Manders' colocalization coefficients [7]. TOS values are rescaled so that −1 corresponds to the minimum possible overlap (anticolocalization), 0 corresponds to the same overlap as would occur by chance (noncolocalization), and 1 corresponds to the maximum possible overlap (colocalization). The default rescaling option is linear because it is easily interpreted, and its value

reflects the fraction between random distribution and the minimum or maximum values (-1 or +1 respectively). For example, a value of 0.5 represents half the maximum possible overlap. EzColocalization also permits logarithmic rescaling (natural log) for users requiring a metric without a discontinuity in the first derivative, but it is harder to interpret than linear rescaling [7]. As mentioned above, TOS is suitable for the analysis of experiments that have non-monotonic relationships, mixed patterns of localization, or unclear localization patterns (**Fig. S2**). TOS can also be used for monotonic relationships including linear correlations, although in such cases it may not be as sensitive or specific as other metrics (*e.g.* PCC).

In summary, PCC is often used for datasets where the reporters have an approximately linear relationship between the pixel values. SRCC and ICQ are commonly used to evaluate whether the signal intensities of the two reporters have a monotonic relationship, and are generally considered more robust to outliers than PCC. Manders' M1 and M2 or TOS are often preferred in cases where there is no clear monotonic localization pattern, or mixed patterns of localization.

6. Colocalization metrics for three reporter channels

The metrics for two channels in EzColocalization are: (i) PCC; (ii) SRCC; (iii) ICQ; (iv) Manders' coefficients; and (v) TOS with linear or logarithmic rescaling. Of these metrics, we extended ICQ, Manders' coefficients and TOS (linear or logarithmic rescaling) to measure colocalization for three reporters, and their derivations are below. PCC and SRCC were not extended for three reporters because their meaning and interpretation becomes much more complicated. Specifically, no single value of PCC or SRCC can represent the standardized covariance. Instead there are multiple values, each of which reports the extent that two channels (independent variables) can predict the signal in the third channel (dependent variable). The first component of principal component analysis (PCA) should be used to measure linearity without assuming dependency of three channels [18]. However, PCA is difficult to interpret in relation to colocalization analysis and therefore was not included [19].

Li's ICQ [3] can be easily expanded to three (or more) channels.

$$\mathrm{ICQ} = \frac{N_{above}+N_{below}}{N_{total}} - 0.5, \qquad \text{Eq. 1}$$

where N_{above} is the number of pixels above the means of all three channels, N_{below} is the number of pixels below the means of all channels, and N_{total} is the total number of pixels. For two channels, ICQ is a crude measure of the fraction of pixels that are on the positive diagonal; that is, it can be interpreted as the fraction of pixels that are broadly consistent with a monotonic increasing relationship. For three channels, ICQ provides a crude measure of whether pixel values tend to increase in all three channels. However, the interpretation of the value is more complicated because of the combinatorics; a pixel may have values above or below the mean in eight possible combinations. A value of −0.25 would be expected if the pixel values have a random distribution, and assuming the median and mean values are approximately equal. In this case, a value >-0.25 may indicate a positive relationship, but it does not exclude the co-presence of a negative relationship. A value of <-0.25 indicates a negative relationship but it does not rule out a positive relationship in a subset of pixels.

The use of Manders' colocalization coefficients for three channels (*i.e.* M1, M2, and M3) has been previously reported [4]. The derivation of Manders' colocalization metrics for more than two channels is straight forward as it simply evaluates the proportion of overlapping signal. However, Manders' colocalization metric are often used with an automated method of threshold selection, such as Costes' method, and these methods typically do not readily extend to three channels [8]. Therefore, EzColocalization users can either select thresholds manually or by using the metric matrix for Manders' colocalization coefficients with three channels. The thresholds are measured as F_T. M_1, M_2, … M_n can be calculated by Eq. 2 where there are at least two reporters:

$$\mathrm{M}_i = \frac{\sum \mathrm{G}_{i,coloc}}{\sum \mathrm{G}_i}, \qquad \text{Eq. 2}$$

where $\mathrm{G}_{i,coloc}$ is the value of each pixel in channel i that is above all thresholds and G_i represents the value of each pixel in channel i that is above the threshold for only channel i. The number of Manders' colocalization coefficients is equal to the number of channels, therefore three values need to be interpreted for three reporter channels. Three values can be difficult to interpret collectively and to compare colocalization between samples. Another challenge is that the interpretation of the Manders' colocalization coefficients depends on the selected thresholds [7].

TOS measures the overlap of the signal above the threshold for each channel accounting for the amount of overlap that would be expected to occur by random chance for different thresholds [7]. One of the first steps in calculating TOS is to determine the number of pixels in each cell that exceed the thresholds for all three reporter channels (A_{coloc}) and the number of pixels that exceed the threshold for one of the reporter channels (A_i, where *i* is the *i*th channel). Dividing the former by the latter is the "observed AO". This calculation, is equivalent to calculating the fraction of pixels in the cell that exceed the thresholds for all three channels (F_{coloc}) divided by the fraction of pixels that exceed the threshold for the chosen channel *i* (F_{Ti}). That is,

$$\text{observed AO}_i = \frac{\mathrm{A_{coloc}}}{\mathrm{A}_i} = \frac{\mathrm{A_{coloc}}/\mathrm{A_{total}}}{\mathrm{A}_i/\mathrm{A_{total}}} = \frac{\mathrm{F_{coloc}}}{\mathrm{F}_{\mathrm{T}i}}, \text{where } i = 1, 2 \text{ or } 3. \qquad \text{Eq. 3}$$

Note: F_{Ti} and F_{coloc} are fractions rather than percentages for all equations in this section, and are defined as greater than zero and less than or equal to one.

The next calculation is the expected AO value assuming uniformly distributed random pixel values. If the pixels above the threshold for the first channel are randomly distributed throughout the cell, then the chance a pixel above the threshold for the second channel overlaps one of the pixels that exceeds the threshold for the first channel, is simply equal to the fraction of pixels above the threshold for the first channel (previously explained elsewhere [7]). Following from this, the chance a pixel that exceeds the threshold for the third channel overlaps a pixel that already exceeds both the first and second channels is simply the product of the fraction of pixels that exceed the first and second reporter channels. Therefore, the

$$\text{expected AO}_i = \frac{F_{T1} \times F_{T2} \times F_{T3}}{F_{Ti}}, \text{where } i = 1, 2 \text{ or } 3. \qquad \text{Eq. 4}$$

The observed AO is divided by the expected AO to generate the “AO ratio”, which accounts for the increase in overlap that occurs with selection of more pixels (*i.e.* greater F_T).

$$\text{AO ratio} = \frac{F_{coloc}}{F_{T1} \times F_{T2} \times F_{T3}}. \qquad \text{Eq. 5}$$

The AO ratio is equal to 1 for cells where the overlap is the same as expected by chance. The value of the AO ratio depends on whether the observed overlap is more or less than expected by chance as well as the selected thresholds. The latter can make interpretation difficult, therefore the AO ratio is rescaled to generate the TOS, which enables easier comparison of analyses with different thresholds.

To rescale the AO ratio, the minimum and maximum value must be determined for the thresholds. The minimum AO ratio can be zero if the sum of the F_T for two channels is less than or equal to 1 (*i.e.* $F_{T1} + F_{T2} \leq 1$). In the case where the first two channels do not overlap, the threshold for the third channel is inconsequential. If there is no overlap of pixels above the thresholds for two channels then there can be no overlap of all three channels, even if all the pixels are selected for the third channel (*i.e.* $F_{T3} = 1$) and consequently the minimum AO ratio would be zero. That is, if $F_{T1} + F_{T2} + F_{T3} \leq 2$, it is possible for the minimum AO ratio to be equal to zero. If $F_{T1} + F_{T2} + F_{T3} > 2$ then overlap of all three channels must occur by at least the amount exceeding 2. In summary,

$$\text{minimum AO ratio} = \begin{cases} \frac{F_{T1} + F_{T2} + F_{T3} - 2}{F_{T1} \times F_{T2} \times F_{T3}}, \text{when } F_{T1} + F_{T2} + F_{T3} > 2 \\ 0, \text{ when } F_{T1} + F_{T2} + F_{T3} \leq 2 \end{cases}. \qquad \text{Eq. 6}$$

The limits of the minimum AO ratio are 0 and 1.

The maximum AO ratio occurs when all three channels maximally overlap, and the maximum amount of overlap can be no more than the minimum F_T. For example, if two channels both have thresholds that select 80% of pixels and the third channel only selects 5% of pixels in the cell, then the maximal overlap of the selected pixels can be no more than 5% of the pixels in the cell; that is, the minimum of the three F_T values.

$$\text{Maximum AO ratio} = \frac{\text{minimum}\{F_{T1}, F_{T2}, F_{T3}\}}{F_{T1} \times F_{T2} \times F_{T3}}. \qquad \text{Eq. 7}$$

The last step in calculating TOS is to rescale the AO ratio using the minimum AO ratio and the maximum AO ratio for the corresponding thresholds as previously reported [7]. A TOS value reflects the fraction of the “distance” between random chance (also known as the null distribution) and the minimum or maximum possible overlap for the thresholds. A positive value indicates colocalization, zero indicates overlap that is no more or less than a random distribution, and a negative value is anticolocalization. For example, 0.5 is halfway between a random distribution and maximum TOS value (half-maximal colocalization for the chosen thresholds) and −0.5 is halfway between a random distribution and the minimum possible TOS value (half-maximal anti-colocalization for the chosen thresholds). It should be noted that the contribution of each channel to the colocalization measurement is not specified in the TOS value. Therefore, anticolocalization may be due to one single channel not overlapping with the other two (as opposed all channels not overlapping).

7. Custom analysis

The Custom subtab in the Analysis tab allows users to perform custom mathematical analysis for all pixel intensity values in selected cells without having to directly modify the code for EzColocalization. In brief, custom written code inserted into the Custom subtab of the plugin uses the same cells or organisms that would be selected by the cell filters (with any alignment used) for non-custom analyses. Each cell's pixel intensity values for each reporter channel are stored in an array, named c1, c2, and c3 for reporter channels 1, 2, and 3 respectively. The order of the pixels within each array is the same; that is, the same index within each array is the same pixel in each channel, and is the intensity value for that channel. The pixel values in the arrays can be analyzed using code written with standard mathematical functions in Java. Selecting the "Resource" button takes the user to a website with a list of operators and functions in Java for mathematical calculations.

8. Supplementary references

1 Ferreira, T. & Rasband, W. *ImageJ User Guide — IJ 1.46.* https://imagej.nih.gov/ij/docs/guide/user-guide.pdf (2012).

2 Thevenaz, P., Ruttimann, U. E. & Unser, M. A pyramid approach to subpixel registration based on intensity. *IEEE Trans Image Process* **7**, 27-41, doi:10.1109/83.650848 (1998).

3 Li, Q. *et al.* A syntaxin 1, Galpha(o), and N-type calcium channel complex at a presynaptic nerve terminal: analysis by quantitative immunocolocalization. *J Neurosci* **24**, 4070-4081, doi:10.1523/JNEUROSCI.0346-04.2004 (2004).

4 Manders, E. M. M., Verbeek, F. J. & Aten, J. A. Measurement of colocalization of objects in dual-colour confocal images. *J Microsc* **169**, 375-382 (1993).

5 Manders, E. M., Stap, J., Brakenhoff, G. J., van Driel, R. & Aten, J. A. Dynamics of three-dimensional replication patterns during the S-phase, analysed by double labelling of DNA and confocal microscopy. *J Cell Sci* **103 (Pt 3)**, 857-862 (1992).

6 Adler, J. & Parmryd, I. Quantifying colocalization by correlation: the Pearson correlation coefficient is superior to the Mander's overlap coefficient. *Cytometry A* **77**, 733-742, doi:10.1002/cyto.a.20896 (2010).

7 Sheng, H., Stauffer, W. & Lim, H. N. Systematic and general method for quantifying localization in microscopy images. *Biol Open* **5**, 1882-1893, doi:10.1242/bio.019893 (2016).

8 Costes, S. V. *et al.* Automatic and quantitative measurement of protein-protein colocalization in live cells. *Biophys J* **86**, 3993-4003 (2004).

9 Harrington, K. I., Stiles, T. S., Venkatraman, L., Prahst, C. & Bentley, K. Functional image processing with ImageJ/FIJI. BioImage Informatics Conference (2015).

10 Barlow, A. L., Macleod, A., Noppen, S., Sanderson, J. & Guerin, C. J. Colocalization analysis in fluorescence micrographs: verification of a more accurate calculation of pearson's correlation coefficient. *Microsc Microanal* **16**, 710-724 (2010).

11 Bolte, S. & Cordelieres, F. P. A guided tour into subcellular colocalization analysis in light microscopy. *J Microsc* **224**, 213-232 (2006).

12 Dunn, K. W., Kamocka, M. M. & McDonald, J. H. A practical guide to evaluating colocalization in biological microscopy. *Am J Physiol Cell Physiol* **300**, C723-742 (2011).

13 Adler, J., Pagakis, S. N. & Parmryd, I. Replicate-based noise corrected correlation for accurate measurements of colocalization. *J Microsc* **230**, 121-133, doi:10.1111/j.1365-2818.2008.01967.x (2008).

14 Sheng, H., Stauffer, W. T., Hussein, R., Lin, C. & Lim, H. N. Nucleoid and cytoplasmic localization of small RNAs in Escherichia coli. *Nucleic Acids Res*, **45**, 2919-2934, doi:10.1093/nar/gkx023 (2017).

15 Adler, J. & Parmryd, I. Colocalization analysis in fluorescence microscopy. *Methods Mol Biol* **931**, 97-109, doi:10.1007/978-1-62703-056-4_5 (2013).

16 Cordelieres, F. P. & Bolte, S. Experimenters' guide to colocalization studies: finding a way through indicators and quantifiers, in practice. *Methods Cell Biol* **123**, 395-408 (2014).

17 McDonald, J. H. & Dunn, K. W. Statistical tests for measures of colocalization in biological microscopy. *J Microsc* **252**, 295-302 (2013).

18 Andrews, D. T., Chen, L., Wentzell, P. D. & Hamilton, D. C. Comments on the relationship between principal components analysis and weighted linear regression for bivariate data sets. *Chemometrics and Intelligent Laboratory Systems* **34**, 231-244, doi:https://doi.org/10.1016/0169-7439(96)00031-7 (1996).

19 Suhr, D. D. Principal Component Analysis vs. Exploratory Factor Analysis. SUGI 30 Proceedings, 203-230 (2005).

9. Supplementary figure legends

Fig. S1. Metric matrices and selected fractions. (**A**) Heat maps showing the intensities of Cy3 and DAPI signal for *sodB::gfp* RNA and DNA respectively in a bacterial cell. The *sodB::gfp* RNA was labeled with Cy3 labeled probes by RNA fluorescence in-situ hybridization. Scale bar is 1 µm. (**B**) Metric matrix with TOS values (linear) for the cell in **Panel A**. Each box in the matrix is the TOS value calculated for the pixels that are above the threshold for each channel. The thresholds are measured as the percentage of pixels with the highest signal for each channel (F_T). For this example, the chosen F_T are the top 100%, 75%, 50% and 25% for Cy3 and the top 100%, 80%, 60%, 40% and 20% for DAPI. The calculated value of TOS is shown for every combination of thresholds and the approximate value is displayed in the bar to the right. The box is colored black when at least one threshold is 100% because in such cases TOS values are not informative; that is, when 100% of pixels are selected for at least one reporter then the overlap with the other channel must always be 100%. Threshold combinations indicated by the purple box and gold dash line box are discussed in **Panel C**. (**C**) Scatterplot of the pixels in the cell in **Panel A**. The purple box has pixels that are both in top 75% and the top 80% of values for Cy3 and DAPI respectively, which are used to calculate the TOS value shown in the purple box in the metric matrix (**Panel B**). The gold dash line box has pixels that are both in top 20% and the top 25% of values for Cy3 and DAPI respectively, which are used to calculate the TOS value shown in the gold box in the metric matrix (**Panel B**).

Fig. S2. Scatterplots identify the relationship between signal intensities. Scatterplots reveal the relationship between the intensities of different reporters, which is important for selecting an appropriate colocalization metric. Three relationships and the recommended metric for measuring colocalization for each are shown (see text of Supplementary Information). The blue line and the circles indicate the hypothetical relationship and hypothetical data points respectively.

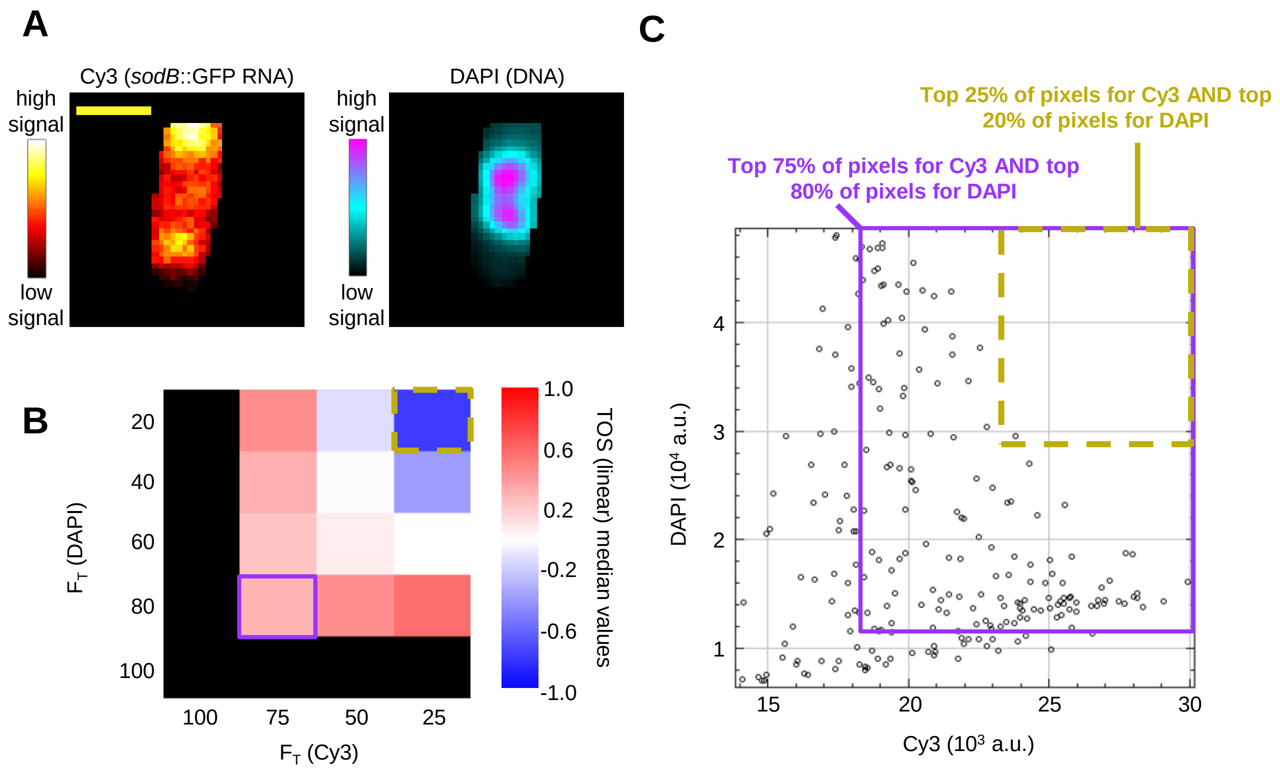

Fig. S1. Metric matrices and selected fractions.

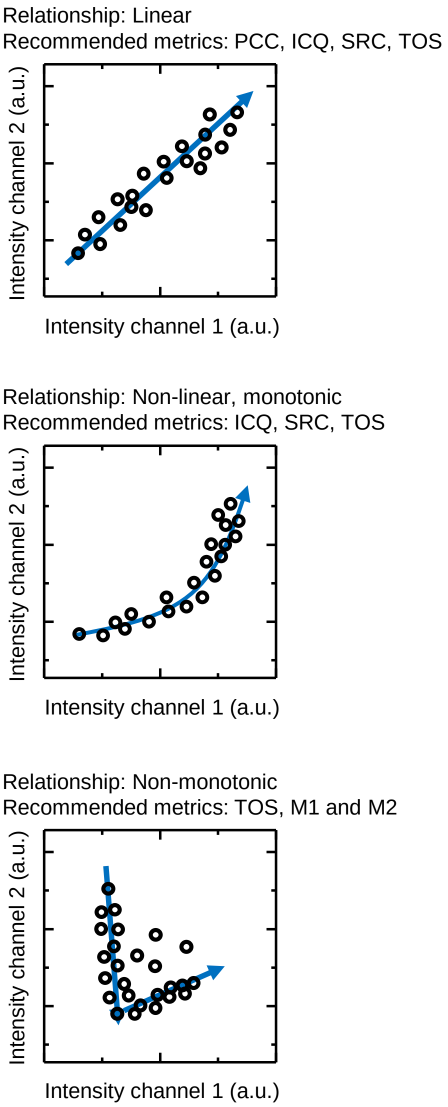

Fig. S2. Scatterplots identify the relationship between signal intensities.

Table S1. Input images and possible outputs from IntracellularJ. * Cell identification channel may be reporter images as discussed in main text. Therefore it is possible to perform all possible analyses with two sets of images (with one set being used as both a cell identification image and a reporter image). # Cell identification images are required to distinguish intracellular and extracellular signal therefore without them any analysis or normalization must be for whole images or image stacks.

Inputs			Outputs						
Cell identification channel*	1 reporter channel	2 or 3 reporter channels	Report of physical features of cells	Visualization: scatterplots, & metric matrices of cell	Visualization: heat maps of cell	Visualization: scatterplots & metric matrices of image or stack	Visualization: heat maps of image or stack	Analysis: measure colocalization in cells	Analysis: measure colocalization in images & stacks #
Yes	Yes	Yes	Yes	Yes	Yes	Yes	Yes	Yes	Yes
Yes	Yes	No	Yes	No	Yes	No	Yes	No	No
Yes	No	No	Yes	No	No	No	No	No	No
No	Yes	Yes	No	No	No	Yes	Yes	No	Yes
No	Yes	No	No	No	No	No	Yes	No	No
No	No	No	No	No	No	No	No	No	No

Table S2. Physical and signal intensity parameters for cell features. *Units for pixel size are arbitrary units unless users set a scale on the images.

Physical (P) or Signal Intensity (S) parameter	Name	Units	Description
P	Area	$pixel^2$*	Number of pixels in a cell.
P	X	pixel*	Average x-coordinate of a cell.
P	Y	pixel*	Average y-coordinate of a cell.
P	Perim.	pixel*	Length of the outside boundary of a cell.
P	Width	pixel*	Width of a cell in the x-axis.
P	Height	pixel*	Height of a cell in the y-axis.
P	BX	pixel*	Top left x-coordinate of the smallest rectangle enclosing a cell.
P	BY	pixel*	Top left y-coordinate of the smallest rectangle enclosing a cell.
P	Major	pixel*	Primary axis of the best fit ellipse for a cell.
P	Minor	pixel*	Secondary axis of the best fit ellipse for a cell.
P	Circ.	unitless	Circularity of a cell calculated by $4 \pi \times$ area $\div$ $perimeter^2$. A value of 1 is a perfect circle and <1 is an ellipse.
P	Angle	degree	Angle between the main axis of an ellipse fit to a cell and x-axis of the entire image containing the cell.
P	Feret	pixel*	Longest possible distance between any two points on a cell boundary.
P	FeretX	pixel*	Starting x-coordinate of the Feret's diameter of a cell.
P	FeretY	pixel*	Starting y-coordinate of the Feret's diameter of a cell.
P	FeretAngle	degree	Angle between a cell's Feret's diameter and its images x-axis.
P	MinFeret	pixel*	Minimum caliper diameter of a cell.
P	AR	unitless	Aspect ratio of a cell calculated by major axis ÷ minor axis.
P	Round	unitless	Roundness of a cell calculated by $4 \times$ Area $\div \pi \times$ major $axis^2$.
P	%Area	unitless	Percentage of pixels in an image, which are included in a cell.
P	Solidity	unitless	Solidity of a cell calculated by its area ÷ area of its convex hull.
S	Mean (Ch. 1), (Ch. 2), or (Ch. 3)	arbitrary	Mean of pixel intensity values for a cell in reporter channels 1, 2, or 3.
S	Mode (Ch. 1), (Ch. 2), or (Ch. 3)	arbitrary	Mode of pixel intensity values for a cell in reporter channels 1, 2, or 3.
S	Median (Ch. 1), (Ch. 2), or (Ch. 3)	arbitrary	Median of pixel intensity values for a cell in reporter channels 1, 2, or 3.
S	Min (Ch. 1), (Ch. 2), or (Ch. 3)	arbitrary	Minimum pixel intensity value for a cell in reporter channels 1, 2, or 3.
S	Max (Ch. 1), (Ch. 2), or (Ch. 3)	arbitrary	The maximum pixel intensity value for a cell in reporter channels 1, 2, or 3.
S	StdDev (Ch. 1), (Ch. 2), or (Ch. 3)	arbitrary	Standard deviation of pixel intensity values for a cell in reporter channels 1, 2, or 3.
S	Skew (Ch. 1), (Ch. 2), or (Ch. 3)	unitless	Skewness of pixel intensity values for a cell in reporter channels 1, 2, or 3.
S	Kurt (Ch. 1), (Ch. 2), or (Ch. 3)	unitless	Kurtosis of pixel intensity values for a cell in reporter channels 1, 2, or 3.
S	RawIntDen (Ch. 1), (Ch. 2), or (Ch. 3)	arbitrary	Sum of all pixel intensity values for a cell in reporter channels 1, 2, or 3.
S	IntDen (Ch. 1), (Ch. 2), or (Ch. 3)	$pixel^2 \times$ arbitrary*	Product of area and average pixel intensity value for a cell in reporter channels 1, 2, or 3.
S	Mean BgndRatio (Ch. 1), (Ch. 2), or (Ch. 3)	unitless	Average pixel intensity value for a cell divided by the average pixel intensity value for all pixels outside of cells in reporter channels 1, 2, or 3. For example, "1-2" for Ch. 1 would select cells with mean pixel intensity one to two fold the mean pixel intensity outside cells for reporter channel 1.
S	Median BgndRatio (Ch. 1), (Ch. 2), or (Ch. 3)	unitless	Median pixel intensity value for a cell divided by the median pixel intensity value for all pixels outside of cells in reporter channel 1, 2, or 3. For example, "1-2" for Ch. 1 would select cells with median pixel intensity one to two fold the median pixel intensity outside cells in reporter channel 1.
